# Oxytocin modulates inhibitory balance in the prelimbic cortex to support social memory consolidation during REM sleep

**DOI:** 10.7150/thno.109104

**Published:** 2025-02-18

**Authors:** Yan-chao Liu, Yu-chen Deng, Zi-tao Zhu, Bo Rao, Hong-lei Shang, Li-ke Wang, Tao Li, Ya-rong Wang, Jian-Zhi Wang, Qing-ping Zhang, Yang Gao, Hai-bo Xu

**Affiliations:** 1 Department of Radiology, Zhongnan Hospital of Wuhan University, Wuhan University, Wuhan, 430071, China.; 2 Second Clinical College, Wuhan University, Wuhan, 430071, China.; 3 Department of Radiology, The Third Affiliated Hospital of Zhengzhou University, Zhengzhou, 450052, China.; 4 Li-Yuan Hospital, Tongji Medical College, Huazhong University of Science and Technology, Wuhan, China.; 5 Department of Pathophysiology, Key Laboratory of Ministry of Education for Neurological Disorders, School of Basic Medicine, Tongji Medical College, Huazhong University of Science and Technology, Wuhan 430030, China.; 6 Hubei Provincial Engineering Research Center of Multimodal Medical Imaging Technology and Clinical Application, Wuhan, 430071, China.; 7 Wuhan clinical research and development center of brain resuscitation and functional imaging, Wuhan, 430071, China.

**Keywords:** oxytocin, social memory consolidation, rapid eye movement sleep, prelimbic cortex, chronic sleep deprivation

## Abstract

**Rationale:** The prelimbic cortex (PrL), enriched with oxytocin (OXT) receptors, plays a critical role in memory consolidation. However, the role of OXT in social memory consolidation within the PrL microcircuit remains poorly understood.

**Methods:** To examine the role of OXT signaling in social memory consolidation, we used OXT biosensors and loss-of-function approaches, including tetanus toxin-mediated silencing of OXT neurons in the paraventricular nucleus (PVN^OXT^), optogenetic inhibition of the PVN^OXT^-PrL pathway during rapid-eye-movement (REM) sleep, and local administration of an OXT receptor antagonist in the PrL. *In vivo* molecular biosensors for vasoactive intestinal peptide (VIP), somatostatin, and presynaptic calcium imaging were employed to assess inhibitory signaling in the PrL microcircuit. Optogenetic activation of the PVN^OXT^-PrL pathway and intranasal OXT were used to evaluate resilience to chronic sleep deprivation-induced social memory deficits.

**Results:** We identified that REM-sleep OXT release via the PVN to PrL pathway supports social memory consolidation. OXT signaling deficiency reduces the activity of VIP and parvalbumin (PV) neurons, thereby disrupting the inhibitory balance between somatic inhibition mediated by PV neurons and dendritic disinhibition mediated by VIP neurons in PrL microcircuits during REM sleep. Chronic sleep deprivation (SD) disrupts OXT release and inhibitory balance, leading to pyramidal neuron hyperactivity and social memory impairments. Notably, REM-sleep-specific activation of the PVN^OXT^-PrL pathway or intranasal OXT restores inhibitory balance and rescues social memory deficits in SD mice.

**Conclusion:** Our results reveal how OXT modulates inhibitory balance in the PrL microcircuit to support social memory consolidation during REM sleep, suggesting potential therapeutic strategies for treating sleep-related memory disorders.

## Introduction

Social memory is vital for survival and reproductive success, enabling individuals to recall past social interactions, identify reliable allies or potential mates, and avoid those who may pose threats or have proven untrustworthy 1. Both non-rapid-eye-movement (NREM) sleep and rapid-eye-movement (REM) sleep are essential for memory consolidation 2, 3. Disturbances in these sleep stages are associated with social memory deficits observed in conditions such as post-traumatic stress disorder (PTSD) 4, autism spectrum disorder (ASD) 5 and Alzheimer's disease (AD) 6. For instance, sleep deprivation (SD) disrupts hippocampal reactivation and replay, processes fundamental to memory formation, leading to pronounced impairments in memory 7. However, the neural microcircuit mechanisms linking sleep disturbances to social memory deficits remain poorly understood.

Reactivation of hippocampal neurons during NREM sleep stabilizes and integrates newly acquired information, playing a pivotal role in memory consolidation 8-10. REM sleep further refines these processes by orchestrating neural population dynamics and enhancing long-term retention 11. Recent studies have highlighted the importance of specific neural circuits in these mechanisms. For instance, disrupting the activity of adult-born neurons in the dentate gyrus during REM sleep impairs memory consolidation 12, and the supramammillary (SuM) nucleus-to-hippocampal CA2 circuit supports REM-dependent social memory 13. Beyond the hippocampus, excitatory pyramidal (PYR) neurons in the prefrontal cortex (PFC) have been implicated in both social memory 14 and REM sleep duration 15. The PFC comprises four major neuronal subtypes: pyramidal (PYR) neurons, vasoactive intestinal peptide (VIP) interneurons, somatostatin (SST) interneurons, and parvalbumin (PV) interneurons 16. PV interneurons mainly exert feed-forward inhibitory effects on PYR somas 17, while VIP interneurons preferentially inhibit SST neurons, which in turn primarily synapse onto the dendritic of PYR neurons 18. During REM sleep, a dynamic balance between somatic inhibition mediated by PV interneurons and dendritic disinhibition mediated by VIP interneurons emerges 19. Additionally, VIP interneurons can directly or indirectly (via SST) modulate PV activity 20, contributing to a complex network of inhibitory and disinhibitory interactions. Despite this intricate interplay, the role of inhibitory balance within PFC circuits in REM sleep-dependent social memory consolidation remains poorly understood.

The prelimbic cortex (PrL), a subregion of the PFC densely populated with oxytocin (OXT) receptors 21, receives projections from oxytocinergic neurons in the paraventricular nucleus (PVN) 22. OXT is a key regulator of social memory in mice 23, 24. Recent advancements in G-protein-coupled receptor activation-based (GRAB) OXT sensors have enabled precise *in vivo* monitoring of OXT release 25. Although both PV and VIP neurons are implicated in memory-related neural dynamics in PFC circuits, the role of their interaction under OXT modulation during REM sleep in the context of social memory remains unclear. Furthermore, SD is known to impair social memory 26 and disrupt cortical circuits, leading to dysfunctions in PYR neurons 27. We hypothesize that OXT modulates REM sleep-related social memory consolidation by regulating the inhibitory balance in PrL circuits, potentially explaining the social memory deficits observed following SD.

In this study, we aimed to elucidate the role of OXT in modulating inhibitory circuits within PrL microcircuits during REM sleep and its contribution to social memory consolidation. By employing OXT, SST, and VIP biosensors, circuit-specific optogenetics, localized OXT receptor antagonists, and presynaptic calcium imaging, we investigated how OXT signaling modulates the balance between somatic inhibition mediated by PV neurons and dendritic disinhibition mediated by VIP neurons. Additionally, we explored how disturbances in REM sleep and OXT signaling, such as those induced by chronic SD, disrupt this balance and impair social memory. By selectively activating the PVN^OXT^-PrL pathway during REM sleep or administering intranasal OXT, we identified potential mechanisms to mitigate social memory deficits. This work provides novel insights into the critical role of OXT in REM sleep-mediated social memory consolidation and highlights its therapeutic potential in addressing memory impairments caused by sleep disturbances.

## Methods

### Animals

We only used male mice in this study because there were significant differences between male and female mice 28 in social competence and social cognition, which would have complicated the analyses in this study. Adult male wild-type C57BL/6J (8-12 weeks, 24-28 g), PV-Cre (C57BL/6) mice (8-12 weeks, 22-24 g), obtained from Beijing Vital River Laboratory Animal Technology Co., Ltd. VIP-Cre (C57BL/6) mice (8-12 weeks, 23-26 g) were obtained from Genepax Biotechnology Co., Ltd. Animals were housed in groups (4 to 5 per cage) at an ambient temperature of 22 ± 0.5 °C, relative humidity of 60 % ± 2%, a 12-hour automatic light-dark cycle (lights on at 7:00 and off at 19:00), and free access to food and water. All experimental protocols were approved by the Experimental Animal Ethics Committee of the Zhongnan Hospital, Wuhan University (Animal Welfare Assurance no. ZN2023198).

### Stereotaxic surgery

Mice were anesthetized by intraperitoneal injection of 1% sodium pentobarbital (75 mg/kg) and placed on a stereotaxic frame (RWD, Shenzhen, China). The scalp was incised with ophthalmic scissors and the cranial window was opened with a dental drill. Using Pump11 Elite Nanomite (70-4507, Harvard Apparatus, USA), 60 nl of virus was injected through a glass micropipette with an opening of approximately 20 μm attached to a 10 μl micro syringe. After injection at a rate of 17 nl/min, the micropipette was left in place for 8 min and then slowly retracted.

To monitor OXT, VIP, and SST in PrL during sleep-wake, the rAAV9-hSyn-OT1.8 (3.08×10^12^ vg/ml, Brain case Co., Ltd., Wuhan, China), rAAV2/9-DIO-VIP1.7 (5.68×10^12^ vg/ml, Brain VTA Co., Ltd., Wuhan, China), rAAV2/9-camkII-SST2.0 (5.24×10^12^ vg/ml, Brain VTA Co., Ltd., Wuhan, China) was injected into the PrL: anteroposterior (AP), 1.95 mm; mediolateral (ML), ±0.30 mm; Dorsoventral (DV), -2.2 mm.

In optogenetic experiments and fiber-photometry experiments, rAAV2/9-DIO-EF1a-hChR2(H134R)-mCherry-WPRE-pA (2.68×10^12^ vg/ml, Brain VTA Co., Ltd., Wuhan, China), rAAV2/9-DIO-EF1a-eNpHR3.0-mCherry-WPRE-pA (1.17×10^12^ vg/ml, Brain VTA Co., Ltd., Wuhan, China), AAV2/9-Ef1a-DIO-NES-jRGECO1a (≥2×10^12^ vg/ml, Brain case Co., Ltd., Wuhan, China) or rAAV2/9-OXT-Cre-WPRE-hGH-pA (5.16×10^12^ vg/ml, purchased from Brain VTA Co., Ltd., Wuhan, China) were injected into the PVN (AP: -0.65 mm, ML: ±0.2 mm, DV: -4.80 mm). To record the activities of PVN^OXT^ neurons that specifically project to PrL, rAAV2/9-OXT-Cre-WPRE-hGH-pA (5.16 ×10^12^ vg/ml, Brain VTA Co., Ltd., Wuhan, China) and rAAV2/9-CAG-DIO-axon-jGCaMP7b (2.30×10^12^ vg/ml, Brain VTA Co., Ltd., Wuhan, China) were injected into the PVN. To inhibit the release of OXT from PVN, rAAV2/5-EF1a-DIO-tettoxicP2A-mcherry-WPREs (2.47×10^12^ vg/ml, Brain VTA Co., Ltd., Wuhan, China) and rAAV2/9-OXT-Cre-WPRE-hGH-pA were injected into the PVN. To record neuron-specific presynaptic membrane Ca^2+^ imaging, rAAV-EF1a-DIO-synaptophysin-jGCaMP7b (5.58 ×10^12^ vg/ml, Brain case Co., Ltd., Wuhan, China) were injected into the PrL in VIP-Cre or PV-Cre mice. 1:2 volume mixtures of Cre-dependent recombinant adeno-associated virus.

### Cannula implantation and intra-PrL infusion

Cannula implantation and local drug application were prepared as previously described 29. Briefly, mice were anesthetized with 1% isoflurane and placed in a stereotactic device (E07370-005, RWD), and maintained at body temperature with a heating blanket. Bilateral implantation of a stainless-steel guide cannula (outer diameter 0.4 mm, inner diameter 0.2 mm) was performed above the PrL region (20° left/right tilt), precisely located at coordinates 1.95 mm anterior to bregma, 1.20 mm lateral to the midline, and 1.75 mm below the pia. To prevent clogging of the guide cannula, a solid stainless-steel wire of 0.18 mm in diameter was inserted, with the prosthesis extending 0.2 mm beyond the guide cannula. The cannula was then firmly fixed to the skull with dental adhesive to ensure stable placement of the cannula. All mice underwent a recovery period of at least one week after implantation of the cannula before subsequent experimental procedures were performed.

Mice were anesthetized with 1% isoflurane and saline or L-368,899 (MedChemexpress, New Jersey, USA), a selective OXTR antagonist, was delivered into the PrL using an injection cannula (outer diameter 0.18 mm, inner diameter 0.09 mm) extending 0.25 mm beyond the tip of the guide cannula. L-368,899 (1.25 mM in 500 nl per side) 30 injected at a speed of 150 nl/min into PrL. L-368,899 is usually effective within 30 min after injection, with effects lasting up to 2-4 h 31, 32. After injection, the cannula was left in place for an additional 6 min to promote drug diffusion.

### OXT administration

Intranasal OXT application provides direct access to the brain through the nasal cavity 33. OXT (MedChemexpress, New Jersey, USA) was dissolved with sterile saline, then aliquoted and stored at -20℃ until use. In brief, 2 mg OXT was dissolved in sterile saline to prepare a stock solution at a concentration of 1 mg/mL. For intranasal administration, the dose was adjusted to achieve 0.1 μg/g body weight 29. For a 20 g mouse, this corresponded to a total dose of 2 μg. To achieve the target dose, the stock solution was diluted to a working concentration of 0.2 μg/μL by performing a 1:5 dilution with sterile saline. Mice were administered 5 μL per nostril (10 μL total), ensuring a final dose of 0.2 μg/μL of the working solution. Control mice received the same volume of saline. To minimize stress responses, mice were acclimatized to a holding position for 3 days before OXT was administered.

### Polysomnographic recording and analysis

Three weeks after virus injection, EEG-EMG electrodes were implanted for mice. The implants consisted of four stainless steel screws (two serving as EEG electrodes and the other two as reference and ground screw, respectively), and two EEG screws were inserted into the frontal lobe area (AP 1.50 mm, ML 1.50 mm) and the lateral parietal lobe area (AP -3.0 mm, ML +2.0 mm). Two EMG electrodes with leads were placed between the neck muscle tissues and the incision was closed. All electrodes are pre-soldered to the mini-pin connector. The EEG and EMG instruments were fixed to the skull with dental cement. One week after postoperative recovery, the mice were attached to recording leads and placed in experimental cages for two days of habituation. The recordings were first scored semi-automatically by 10 s epochs for the wake, NREM sleep and REM sleep. We defined wake as unsynchronized EEG and high levels of EMG activity. NREM sleep was defined as synchronized, high amplitude, low frequency (0.5-4 Hz) EEG. Micro-arousals during NREM are defined according to previous literature 34. REM sleep was defined as having a significant theta rhythm (4-9 Hz) and no EMG activity.

### Fiber recordings and data analysis

The fiber photometry system (Thinker Tech, Nanjing, China) was used to monitor calcium dependent fluorescence (jGCaMP7b and jRGECO1a) and calcium inde-pendent fluorescence (GRAB_OXT1.8_, GRAB_VIP1.7_, and GRAB_SST2.0_ biosensor). In this system, a 405 nm channel effectively eliminates motion noise, a beam of 470 nm laser light excited jGCaMP7b or biosensors (GRAB_OXT1.8_, GRAB_VIP1.7_ and GRAB_SST2.0_), and a 580 nm laser light excited jRGECO1a to detect changes in fluorescence signals. The laser power at the tip of the optical fiber was adjusted to 10∼20 μW (green laser) and 50 uW (red laser) to minimize bleaching. Recordings were performed with the software based on LabVIEW (Thinkertech, Nanjing Bioscience Inc., China) at 30 Hz. All fluorescence signals, EEG/EMG signals and behavioral videos were synchronized offline with event markers. Analysis was performed in MATLAB R2023a (MathWorks) using custom scripts. Z score was then calculated from the detrended signal F as Z score = (F-mean (F_baseline_))/s.d.(F_baseline_), where F_baseline_ was the baseline fluorescence signal. For the sleep-wake analysis, we recorded data for 4-5 h per mouse. For the sleep-wake state transition analyses, we determined the time points of state transition and aligned z score around these times (± 10 s).

### Calculation of Ca^2+^ fluorescence strength, fluorescence power

To assess neuronal activity, we measured Ca^2+^ fluorescence intensity and fluorescence power during the 30 s preceding REM sleep, as REM episodes in C57BL/6 mice typically last for 30-60 s 35. We processed them using a MATLAB code (see Supplementary file: Naming with MATLAB code). Higher fluorescence signal strength may indicate stronger synaptic inputs, increased neuronal excitability, or synchronized discharges among neurons. Fluorescence Power, typically calculated from the signal power spectral density, represents the energy distribution across frequency bands and quantifies the oscillatory nature of neuronal discharge activity.

### Electrophysiological recording

The following formulation scheme is similar to previous reports 36. Mice were anesthetized with sodium pentobarbital and perfused with ice-cold sucrose artificial cerebrospinal fluid (ACSF), saturated with 95% O_2_ and 5% CO_2_ (PH 7.3), containing (in mM): 2.5 KCl, 213 sucrose, 0.4 ascorbic acid, 1.25 NaH_2_PO_4_, 10 glucose, 26 NaHCO_3_, 3 MgSO_4_, 2 Na-pyruvate and 0.1 CaCl_2_. Brains were rapidly removed and sectioned into 320 μm coronal slices using a vibrating microtome (VT1200, Leica, Germany) in ice-cold ACSF. Slices were transferred to recording ACSF (in mM): 1.25 NaH_2_PO_4_, 126 NaCl, 26 NaHCO_3_, 2 CaCl_2_, 25 glucose, 2.5 KCl and 1.0 MgSO_4_. Slices were then recovered at 32°C for 30 min and maintained at room temperature for an additional 30 min before recording.

During recordings, slices were submerged in a chamber and super fused with warm ACSF (30-32°C) at 2 mL/min. PVN regions were localized using low-power fluorescence microscopy and OXT neurons were identified under high-power fluorescence microscopy. Patch electrodes (4-6 MΩ) were filled with an internal solution containing 105 mM potassium gluconate, 30 mM KCl, 4 mM ATP-Mg, 10 mM phosphocreatine, 0.3 mM EGTA, 0.3 mM GTP-Na, and 10 mM HEPES (pH 7.3, 285-300 mOsm). Electrophysiological recordings of cell-attached and whole-cell current-clamp and voltage-clamp (at -70 mV) were performed at 30-32°C using a MultiClamp 700B amplifier (Axon Instruments). Signals were filtered at 4 kHz and digitized at 10 kHz using a DigiData 1440A (Axon Instruments). Data acquisition and analysis were performed with pClamp 10.3 software (Axon Instruments).

### Chronic sleep deprivation

SD was achieved using the automated Sleep Deprivation System (Shanghai Xinruan Information Technology Co., Ltd., Shanghai, China). Animals were kept individually in the apparatus with fresh bedding, food and water for at least 24 h before the start of the experiment and returned to the home cage daily after SD. To induce chronic SD, the bar was rotated continuously (approximately 3 rpm/min) by a motor for 4h daily during the early light phase (11:00 a.m. to 15:00 p.m.). The bar was randomly reversed in a rotational direction to prevent the test mouse from acquiring brief sleep periods because accustomed to this kind of rotation mode. A trained experimenter visually verified that the bars were rotating at all times and that the mice did not use any alternative strategies to sleep.

### Optogenetic stimulation

In optogenetic manipulation experiments, optical fibers (fiber core: 200 μm, numerical aperture: 0.37, Inper, China) were implanted above the PrL. For optogenetic inhibition, fibers were bilaterally implanted at a 20° left/right tilt, whereas for optogenetic activation, fibers were unilaterally implanted vertically. Before the testing day, each mouse was connected to an optical fiber patch cord (4 m long, 200 μm diameter, Inper, Hangzhou, China), which allowed free movement. The patch cord was connected to another fiber optic patch cord (Inper, China), and this assembly was linked to an intelligent optogenetic stimulation system (Thinkertech, Beijing, China) capable of generating 473 nm blue light or 593 nm yellow light.

To ensure consistent stimulation, the optical signal power was calibrated, with the fiber optic cable emitting 8-12 mW at the tip. Importantly, photoinhibition was not applied throughout the entire 4-hour sampling interval. Instead, optogenetic manipulation was applied exclusively during REM sleep episodes. The average light application time per REM episode was less than 70 s. In addition, a similar suppression scheme has been applied in previous 19, 37.

### Two-choice social novelty test

All behavior was monitored with a camera (VisuTrack, Xinruan, Shanghai, China) placed on the top of the open-topped box. We considered the entry of the subject mice within 3 cm of the edge of the cage to be valid exploration (excluding the time taken by the mice to climb to the top of the cage, which they would have spontaneously climbed down). We based our experimental design on previously reported methods 13, 38, 39, and on practical needs. Briefly, a square open-topped box (54 cm ×26 cm × 40 cm) with two 10 cm diameter cage (square-shaped) on opposite sides was used to hold the stimulus mouse. The test involved three phases: habitation, training, and testing. The task was carried out at daylight. Habituation (the day before training): subject mice were allowed to freely explore the arena containing two empty cages for 10 min. Training phase: a stimulus mouse (novel mice) was put into the left or right cage (systematically alternated), another one left empty, and the subject mouse was allowed to freely explore the arena for 10 min. Mice were immediately returned to their original cages and EEG-EMG recordings were monitored to manually identify REM episodes over the subsequent 4 h (social memory consolidation often occurs within the first few hours after memory encoding 12). In the mCherry-REM and eNpHR-REM groups, yellow light was continuously delivered to the axonal terminals of PVN-to-PrL projecting neurons via fiber ferrules from the onset of REM sleep until its termination. In contrast, in the eNpHR-NREM and eNpHR-wake groups, yellow light was applied during NREM sleep and wake, respectively. The total duration of light delivery in these groups matched that of the eNpHR-REM group, but the stimulation was evenly distributed across a 4-hour period and applied randomly. Testing phase: a novel mouse was randomly placed in either the left or right cage, a familiar mouse was placed in the other cage, and then the subject mouse was again allowed to explore freely for 10 min. The social preference index was calculated using the equation: Social preference index = (time for novel mouse - time for familiar mouse or empty cage) / (time for novel mouse + time for familiar mouse or empty cage).

### Immunohistochemistry

Mice were deeply anesthetized with sodium pentobarbital, followed by intracardiac perfusion with saline via the left ventricle, and then fixed in a fixative containing 4% paraformaldehyde (PFA). The brains were removed and fixed in 4% PFA for 12 h and then cryoprotected in sucrose solution (30% sucrose in PBS) at 4 °C. The brains were then sectioned in 40 μm coronal sections at -23 °C on a freezing microtome (CM1950, Leica, Germany). For immunostaining, the floating sections were incubated in blocking buffer (P0260, Beo Tianmei, China) for 40 min at room temperature, and then incubated overnight at 4 °C with primary antibody diluted in primary antibody diluent (P0262, Beo Tianmei, China).The following primary antibodies were used: anti-Oxytocin (1:500, EPR20973, abcam, USA), anti-CamKII (1:500, 3362, Cell signaling, USA), anti-GAD67 (1:500, MAB5406, Sigma, USA), anti-Parvalbumin (1:500, SAB4200545, Sigma, USA). The next day, sections were washed with PBS and incubated with secondary antibodies (Donkey rabbit-mouse Alexa 546, 1:800, GB21303, Service bio or Donkey anti-mouse Alexa 546, 1:800, GB21301, Service bio, USA).

Following the completion of all behavioral tests, the mice were immobilized to confirm the placement of the cannula and the accuracy of drug injections. To verify the locations of the virus expression and optical fiber placement, mouse brains were processed as previously described. Whole mouse brains were sectioned at a thickness of 40 μm. Finally, sections containing traces were rinsed 3 times with PBS and then immersed in DAPI for 20 min at room temperature. Finally, the sections were mounted on slides and covered with coverslips. High-resolution fluorescence images were obtained on a confocal microscope (SP8, Leica, Germany). Digital images were processed using image processing software (image J) to minimize brightness and contrast adjustments.

### Statistical analysis

All data were presented as mean ± standard error of the mean (SEM). Two-tailed paired and unpaired Student's t-tests, one-way ANOVAs were used. Significant effects in ANOVA were followed by Bonferroni's post hoc multiple comparison tests. *P* < 0.05 was considered statistically significant. Post hoc significance values were set as *P < 0.05, **P < 0.01 and ***P < 0.001. All statistical tests used are indicated in the figure legends. The data were analysed and figures were drawn using GraphPad Prism 10.0 software.

## Results

### OXT signaling in PrL was required in REM sleep and social memory

**Figure 1:**
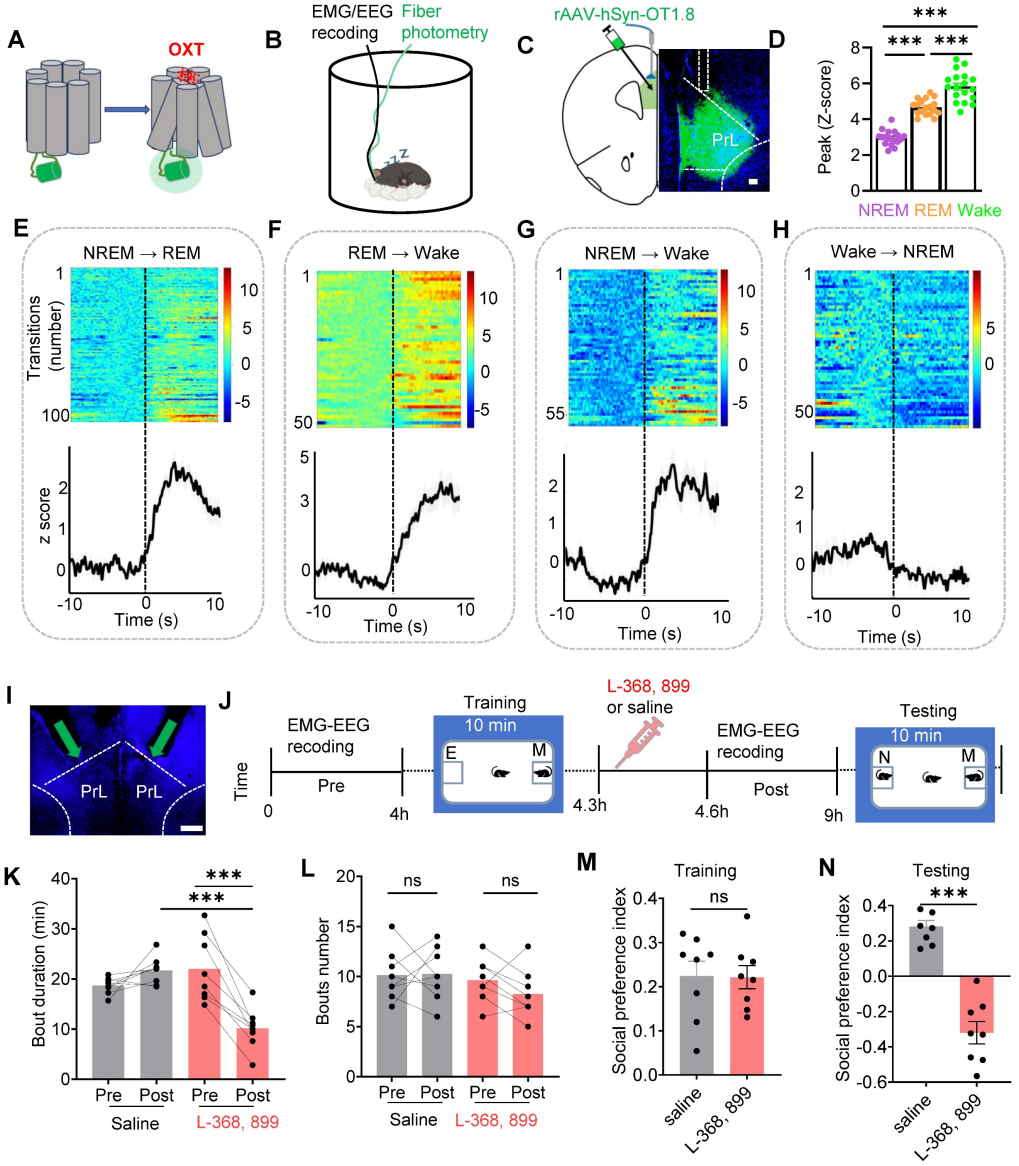
** OXT release in the PrL across sleep-wake states and effects of local OXT receptor antagonists on REM sleep and social memory.** (**A**) Schematic diagram of the action principle of OXT-specific biosensors, GRAB_OXT1.8_. Conformational and fluorescence changes occur when the OXT receptor expressing the fluorescent protein binds to the OXT neurotransmitter. (**B**) Diagram showing fiber photometry and EEG-EMG recording. (**C**) Illustration of the unilateral viral infection site for OXT biosensor (rAAV-hSyn-OXT1.8) expression in unilateral PrL. Scale bar, 200 µm. (**D**) Peak fluorescence of OXT biosensor recordings in the states of REM sleep, NREM sleep and Wake. n = 18, three sessions per mouse from 6 mice; ***p < 0.001, as determined by One-way ANOVA. (**E to H**) Fluorescence signal of OXT biosensor transformation aligned to sleep-wake state transitions. NREM to REM, n = 100; REM to Wake, n = 51; NREM to Wake, n = 59; Wake to NREM, n = 54; recording from 6 mice. The data are presented as the mean (black trace) ± SEM (gray shading). (**I**) Representative image of guide cannula placement above the PrL for administration of L-368,899 (OXT receptor antagonist) or saline. 20° left/right tilt, with the injection needle extended 0.2 mm beyond the guide cannula tip, marked by the green arrow. (**J**) Experimental timeline for cannula administration of the OXT receptor antagonist (L-368,899) or saline, followed by two-choice social novelty test. E, empty; M, mice; N, novel mice; F, familiar mice. (**K and L**) Comparison of REM sleep bout duration and count over 4 h pre- and post- OXT receptor antagonist or saline administration. n = 8 mice per group, ns, *p* > 0.05, ***p < 0.001, as determined by paired and unpaired t-test. (**M and N**) L-368,899-treated mice displayed social memory impairment assessed by two-choice social novelty test. Social preference index was measured in training (M) and testing (N) phase, respectively. n = 8 per group, ns, *p* > 0.05, ***p < 0.001, as determined by unpaired t-test. The data above are presented as the mean ± SEM.

To record the real-time activity of OXT neuropeptide in the PrL during sleep-wake cycles in mice, we employed a novel genetically encoded sensor, GRAB_OXT1.8_, to dynamically record OXT activity in real time (Figure 1A). Conformational changes in G protein-coupled receptors (GPCRs) upon ligand binding, including those induced by the OXT neuropeptide, are linked to shifts in fluorescent protein signals, enabling the detection of neurotransmitter release via fluorescence imaging 25. To simultaneously monitor electroencephalogram (EEG), electromyogram (EMG), and OXT release in the PrL of freely moving mice, we implanted EMG-EEG electrodes and optical fibers targeting the PrL in wild-type (WT) mice (Figure 1B-C). Our results showed that OXT release in the PrL was higher during rapid-eye-movement (REM) sleep and wake compared to non-REM (NREM) sleep (Figure 1D). Notably, OXT levels increased before transitions from NREM to REM (Fig. 1E), REM to wake (Figure 1F), and NREM to wake (Figure 1G). In contrast, no significant changes were observed during wake to NREM transitions (Figure 1H).

To determine whether OXT directly affects social memory, we locally infused either an OXT receptor antagonist (L-368,899, 1.25 mM, 500 nl/side) or saline into the PrL of WT mice (Figure 1I). Subsequently, we conducted a social novelty preference test, a well-established behavioral paradigm 13 to assess the influence of local L-368,899 on sleep structure and social memory. During the training phase, mice explored a chamber containing a novel conspecific alongside an empty chamber for 10 mins. Following this training, the mice received either L-368,899 or saline injections and were then returned to their cages for EEG-EMG recordings. After 4 h, the subject mice were reintroduced to a familiar mouse and a novel mouse for another 10-minute session (Figure 1J). Typically, mice exhibit a strong preference for interacting with novel conspecifics 40. Our results revealed that the L-368,899 group exhibited a significant reduction in REM sleep duration, but the number of REM episodes was not reduced, compared to the saline group (Figure 1K-L). No significant changes were observed in wake or NREM sleep (Suppl Figure S1A-B). Furthermore, L-368,899-treated mice displayed marked impairments in social memory (Figure 1M-N). These findings show that OXT is involved in REM sleep, and that local antagonist of OXT receptors in the PrL reduces REM sleep duration and impairs social memory.

### Dysfunction of PVN^OXT^ neurons impaired OXT release in the PrL, REM sleep and social memory

To confirm that PVN is the primary source of OXT secretion in PrL, we optogenetically activated PVN^OXT^ neurons using rAAV-OXT-Cre and Cre-inducible ChR2 in WT mice. OXT release in the PrL was monitored via the GRAB_OXT1.8_ biosensor, showing a significant increase in OXT fluorescence upon activation (Suppl Figure S2A-D) as expected.

**Figure 2:**
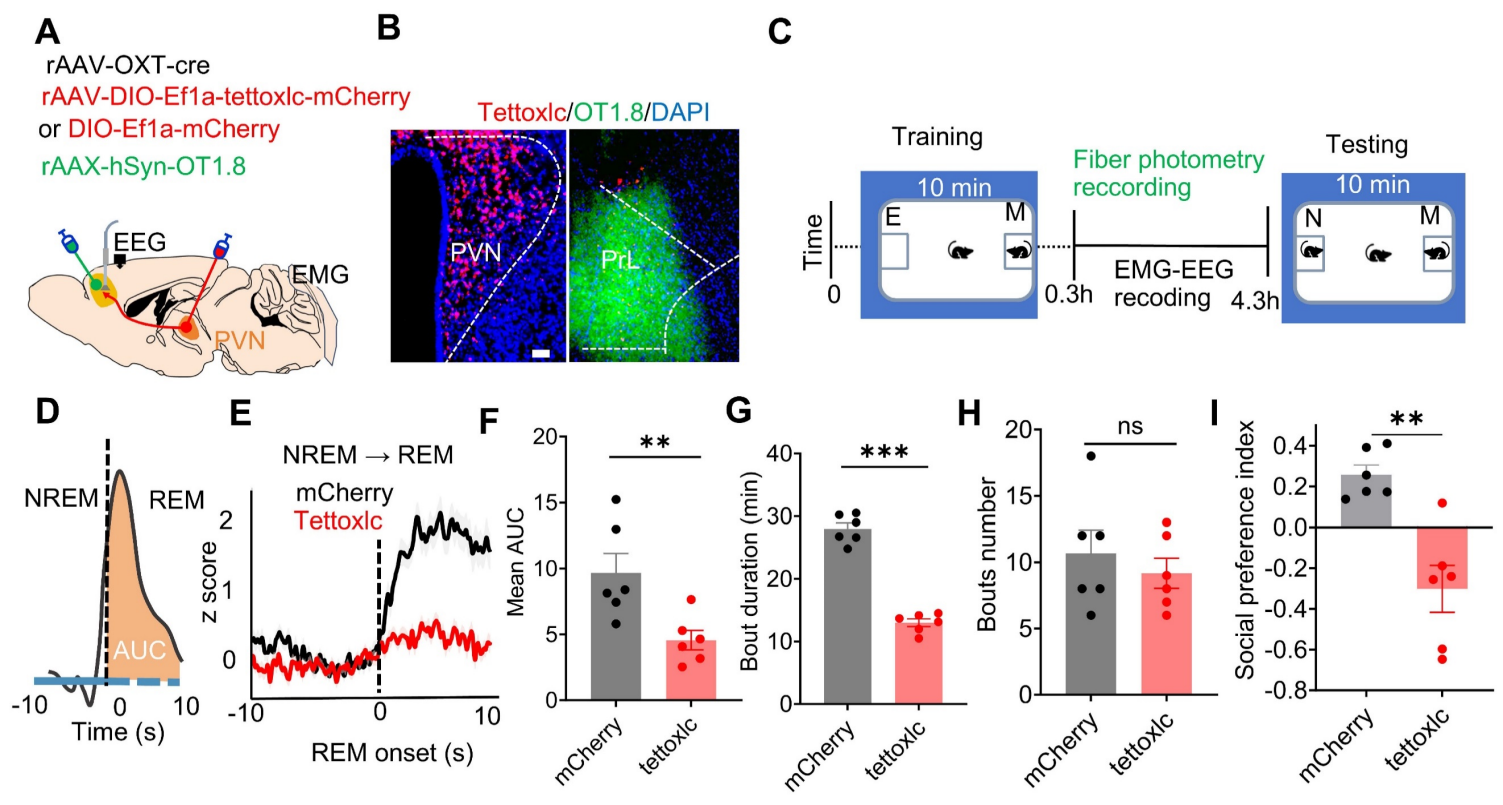
** Dysfunction of PVN^OXT^ neurons impaired OXT release in PrL, REM sleep and social memory.** (**A**) Schematic showing rAAV-OXT-cre, rAAV-DIO-Ef1a-tettoxlc-mCherry or rAAV-DIO-Ef1a-mCherry injection in bilateral PVN and OXT biosensor recording in unilateral PrL setup. (**B**) Representative images of PVN^OXT^ neurons expressing tettoxlc and PrL neurons encoding the GRAB_OXT1.8_ biosensor. (**C**) Experimental timeline outlines the two-choice social novelty test, fiber photometry, and EEG-EMG recordings. E, empty; M, mice; N, novel mice; F, familiar mice. (**D**) Calculation of the area under the curve (AUC) for OXT biosensor fluorescence is presented for the first 10 s of REM sleep. (**E**) Average OXT biosensor fluorescence transients from NREM to REM in mCherry (black trace) and tettoxlc (red trace) groups. n = 100 transitions recording from 6 mice in mCherry group; n = 72 transitions recording from 6 mice in tettoxlc group. The data are presented as the mean ± SEM. (**F**) Comparison of mean AUC for OXT biosensor fluorescence between mCherry and tettoxlc groups n = 6 mice per group; **p < 0.01, as determined by unpaired t-test. (**G and H**) Comparison of REM sleep duration and bout count over a 4-hour period between mCherry and tettoxlc groups. n = 6 mice per group; ***p < 0.001, ns, *p* > 0.05,as determined by unpaired t-test. (**I**) Social memory performance was compared between mCherry and tettoxlc groups. n = 6 mice per group; **p < 0.01, as determined by unpaired t-test. The data above are presented as the mean ± SEM.

To investigate whether dysfunctional PVN^OXT^ neurons affect OXT release in the PrL and social memory, we selectively inhibited PVN^OXT^ neurons using tetanus toxin-mediated damage (Figure 2A-C). Tettoxlc, a neurotoxin produced by Clostridium tetani, enzymatically cleaves synaptic vesicle proteins, impairing vesicle fusion with the presynaptic membrane 41. We injected AAV-OXT-Cre and Cre-inducible tettoxlc into the bilateral PVN of WT mice to inhibit OXT release, while GRAB_OXT1.8_ was injected into the unilateral PrL to monitor OXT release across sleep-wake cycles (Figure 2A- C). Mice in the tettoxlc group showed reduced OXT release in the PrL during REM sleep and wake (Suppl Figure S2E). Additionally, OXT release in this group was reduced during NREM to REM (Figure 2D-F), NREM to wake, and REM to wake transitions, while no significant differences were observed during wake to NREM transitions (Suppl Figure S2F-H). OXT deficiency also altered sleep architecture, increasing wake and reducing the duration of both REM and NREM sleep, although the frequency of sleep episodes remained unaffected (Figure 2G-H; Suppl Figure S2I-J). Furthermore, Tettoxlc-treated mice exhibited deficits in social memory (Figure 2I). These findings indicate that dysfunction of PVN^OXT^ neurons reduce OXT release in the PrL, adversely impacting both sleep architecture and social memory.

### Silencing of PVN^OXT^-PrL pathway during REM sleep impaired social memory consolidation

**Figure 3:**
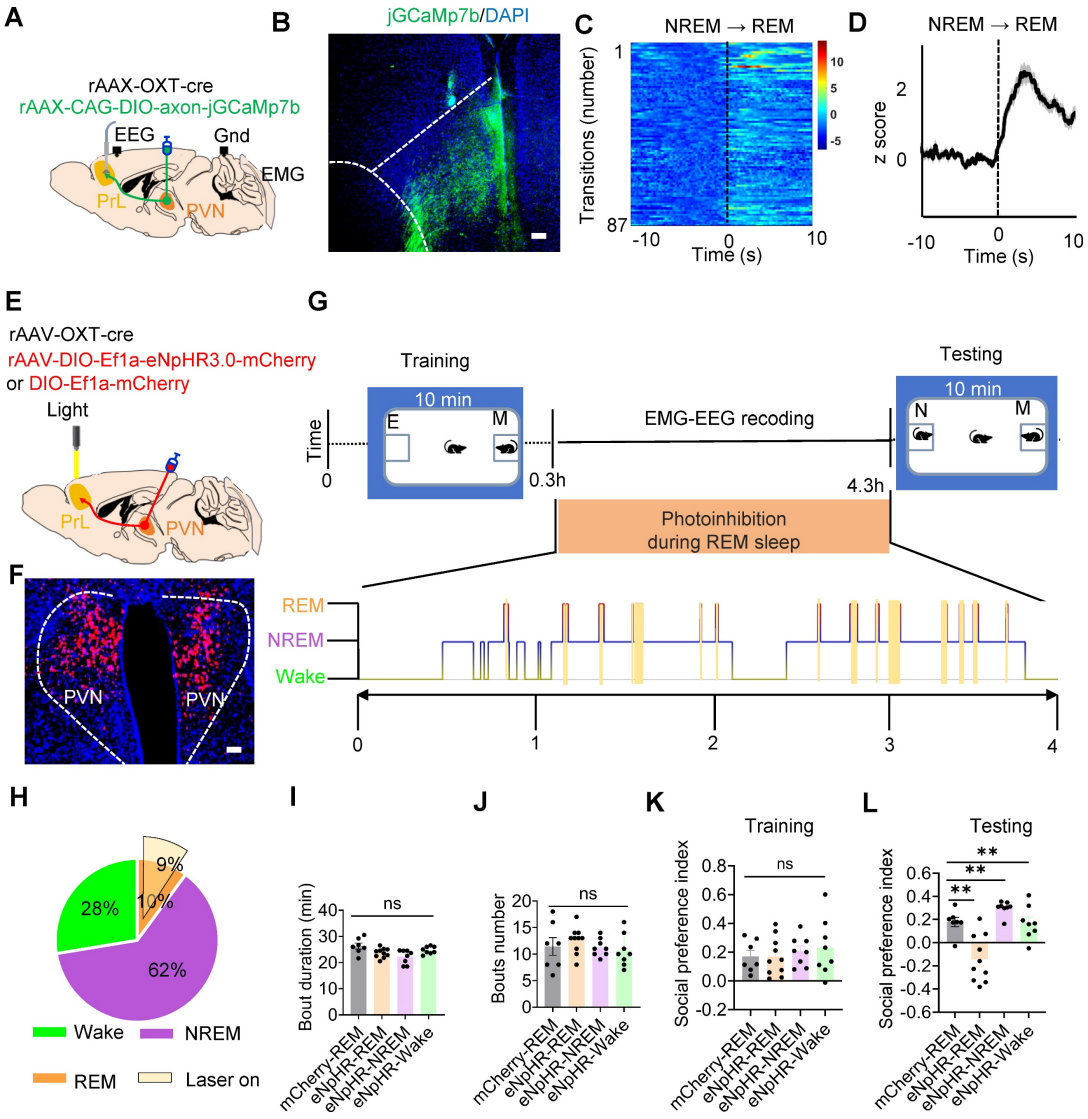
** Silencing of the PVN^OXT^-PrL pathway during REM sleep impaired social memory.** (**A**) Schematic of mixed virus (rAAV-OXT-Cre and rAAV-DIO-axon-jGCaMP7b) injection in unilateral PVN, calcium imaging in PrL, EEG recording in cortex and EMG recording in neck muscles. (**B**) Post-hoc histological verification of jGCaMP7b expression and fiber placement in the PrL (**C and D**) Fluorescence signal of calcium imaging in PrL aligned to NREM to REM transitions. The data are presented as the mean (black trace) ± SEM (gray shading). n = 87 transitions recording from 4 mice. (**E**) Diagram showing bilateral viral injection site of rAAV-OXT-cre, rAAV-DIO-eNpHR3.0-mCherry/rAAV-DIO-mCherry in bilateral PVN, accompanied by the optical fiber implanted above PrL, EEG recording in cortex and EMG recording in neck muscles. (**F**) Representative image of viral expression (rAAV-OXT-cre and rAAV-DIO-eNpHR3.0-mCherry) within the bilateral PVN. (**G**) Protocol outline for optogenetic manipulation during REM sleep and two-choice social novelty test. E, empty; M, mice; N, novel mice; F, familiar mice. (**H**) Total duration of sleep-wake state during optogenetic manipulation in PVN^OXT^-PrL circuit during REM sleep. **(I and J)** Optogenetic inhibition of the PVN^OXT^-PrL pathway during REM sleep, NREM sleep, or wake had no significant effect on the duration (I) or number bouts (J) of REM sleep. mCherry-REM group, n = 7 mice; eNpHR-REM group, n = 10 mice; eNpHR-NREM and eNpHR-Wake groups, n = 8 mice each; ns, *p* > 0.05, as determined by unpaired t-test. **(K and L)** Optogenetic inhibition of the PVN^OXT^-PrL pathway during REM sleep significantly impaired social memory, while inhibition during NREM sleep or wake had no effect. The two-choice social novelty test was used to assess the social preference index during the training phase (K) and the testing phase (L). mCherry-REM group, n = 7 mice; eNpHR-REM group, n = 10 mice; eNpHR-NREM and eNpHR-Wake groups, n = 8 mice each; unpaired t-test, **p < 0.01, ns, *p* > 0.05. All data are presented as mean ± SEM.

We then evaluated whether PVN^OXT^-PrL pathway modulates social memory consolidation within REM sleep. We used a circuit-specific fiber photometry method 42 in freely moving WT mice with simultaneous EEG and EMG recordings. By locally injecting rAAV-OXT-Cre and a Cre-inducible virus (AAV-CAG-DIO-axon-jGCaMP7b) into the unilateral PVN, enabling the expression of the Ca^2+^ sensor in PVN^OXT^ axon (Figure 3A). Three weeks post-injection, we implanted an optical fiber above the PrL to monitor axonal projection activity from PVN^OXT^ neurons (Figure 3B). Notably, Ca^2+^ activity in PVN^OXT^ axonal terminals increased significantly during NREM to REM sleep transitions (Figure 3C-D), indicating heightened PVN^OXT^-PrL pathway activity associated with REM sleep.

Given the strong activation of the PVN^OXT^-PrL during REM sleep, then we selectively silenced this projection during REM sleep and assessed its impact on social memory consolidation. We injected both rAAV-OXT-Cre along with either Cre-dependent rAAV-eNpHR3.0 or rAAV-mCherry into the bilateral PVN of WT mice (Figure 3E-F) and performed two-choice social novelty tests to determine the role of REM-specific PVN^OXT^-PrL pathway activity in social memory (Figure 3G). Optogenetic silencing was applied during approximately 90% of cumulative REM sleep in the eNpHR and mCherry groups (Figure 3H). During the initial 4 h of memory consolidation following training, optogenetic stimulation was applied using yellow light through an implanted optical fiber to the PVN^OXT^-PrL pathway in the mCherry-REM or eNpHR-REM groups. Yellow light was delivered immediately upon the onset of REM sleep and continued until the end of each REM episode. A second control group of mice expressing eNpHR3.0 in PVN^OXT^ neurons (eNpHR-NREM and eNpHR-wake) underwent the same procedure as the mCherry-REM or eNpHR-REM groups, except that yellow light was delivered during NREM sleep or wake episodes, for a similar duration as in the REM groups. Notably, REM sleep/NREM sleep/Wake-specific silencing of the PVN^OXT^-PrL pathway did not alter overall sleep architecture, as indicated by six parameters: total REM sleep duration (Figure 3I), number of REM sleep episodes (Figure 3J), total NREM sleep duration, number of NREM bouts, total wake time, and number of wake bouts (Figure S3). Unlike local application of the OXT receptor antagonist in the PrL (Figure 1K), photoinhibition of the PVN^OXT^-PrL pathway during REM sleep/NREM sleep/Wake did not cause prolonged disruption of OXT signaling of mice. During the training phase, there was no significant difference in the social preference index among the mCherry-REM, eNpHR-REM, eNpHR-NREM, and eNpHR-wake groups (Figure 3K). In the testing phase, only the eNpHR-REM group exhibited impaired social memory (Figure 3L), suggesting that silencing the PVN^OXT^-PrL pathway during REM sleep specifically disrupts social memory consolidation. These results establish the critical role of the PVN^OXT^-PrL pathway in supporting social memory during REM sleep.

### OXT modulated inhibitory balance in PrL microcircuits during REM sleep for social memory consolidation

**Figure 4:**
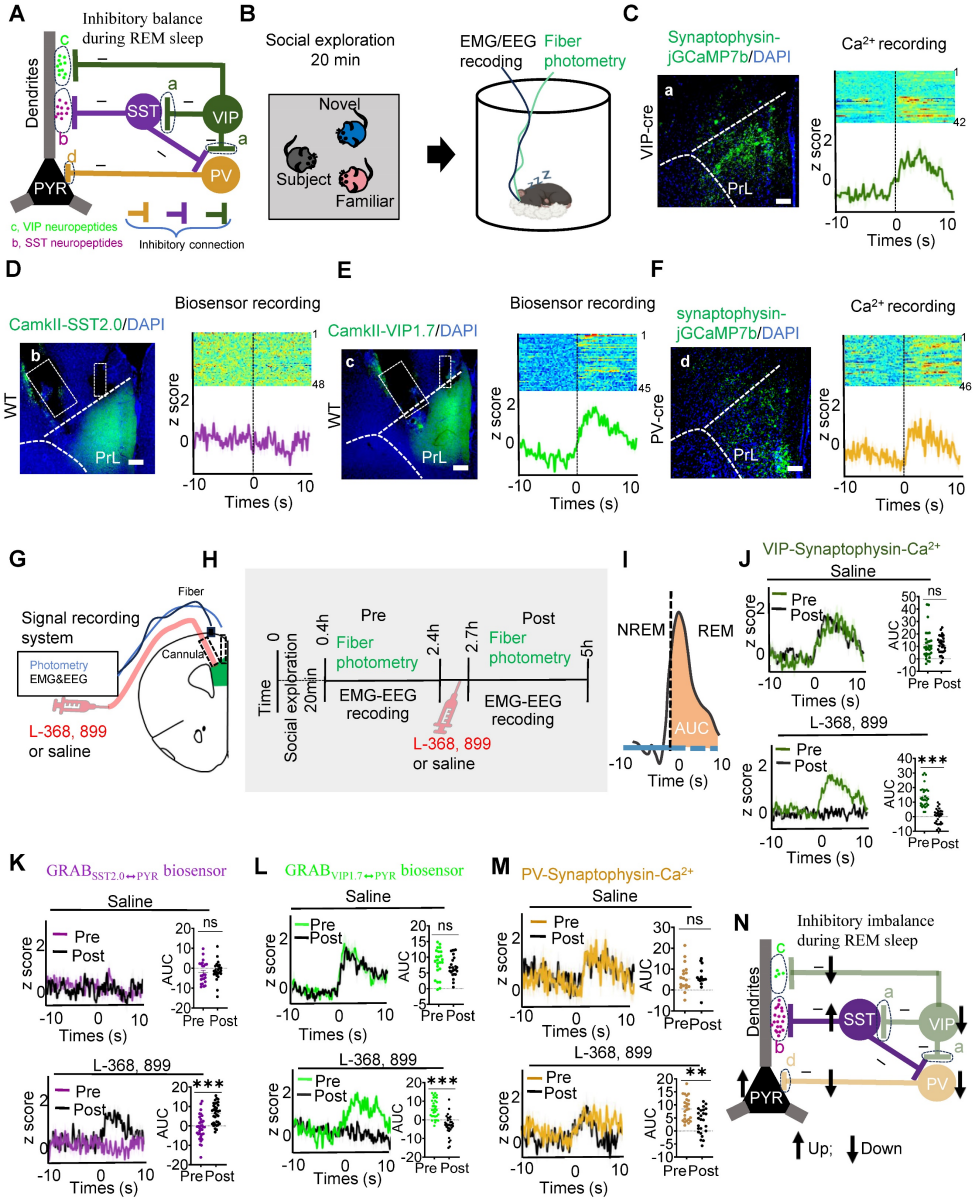
** OXT modulated inhibitory balance in PrL microcircuits during REM sleep.** (**A**) Working model illustrating the inhibitory balance in PrL circuits during REM sleep related to social memory consolidation. Three distinct types of inhibitory balance in this model: (1) VIP and SST neuropeptides maintain dendritic inhibition in PYR neurons during REM sleep; (2) VIP and SST interneurons modulate PV activity, stabilizing somatic activity in PYR neurons; and (3) a dynamic balance exists between PV-mediated somatic inhibition and VIP-mediated dendritic disinhibition during REM sleep. a, Presynaptic membrane of VIP interneurons; b, SST neuropeptides that act specifically on PYR neurons; c, VIP neuropeptides that act specifically on PYR neurons; d, Presynaptic membrane of PV interneurons. **(B)** Social memory consolidation paradigm. The subject mice explored a novel and familiar conspecific for 20 min (left); EMG, EEG, and fiber photometry data were recorded during sleep in their home cage (right). **(C-F)** Injection site**s** of rAAV-DIO-synaptophysin-jGCaMP7b in the unilateral PrL of VIP-Cre mice (C) or PV-Cre mice (F). GRAB_SST⬌PYR_ biosensor (D, rAAV-camkII-SST1.8) and GRAB_VIP⬌PYR_ biosensor (E, mix virus injection of rAAV-CamkII-cre and rAAV-DIO-VIP1.7) expression in the unilateral PrL of WT mice. Scale bar = 200 µm. VIP/PV synaptophysin-Ca^2+^ (C and F) and SST/VIP biosensor (D and E) recording in the PrL during NREM-to-REM sleep transitions. The colormaps represent the trial-by-trial data, while the traces below represent the averaged data. These data were collected prior to the application of L-368, 899 or saline. Green, VIP synaptophysin-Ca^2+^ (a); purple, VIP neuropeptides (b); cyan, SST neuropeptides(c); brown, PV synaptophysin-Ca^2+^(d). (**G**) Diagram of cannula placement and setup for fiber photometry, EMG and EEG recordings. (**H**) Timeline showing administration of L-368, 899 and saline. (**I**) Calculation of the AUC for synaptophysin-Ca^2+^ or biosensor fluorescence is presented for the first 10 s of REM sleep. (**J to M**) SST/VIP biosensor and VIP/PV synaptophysin-Ca^2+^ recording before and after saline or L-368, 899 application. (**J**) Synaptophysin-Ca^2+^ recording in VIP interneurons before and after saline (upper; Pre, n = 30; Post, n = 30; recording from 6 mice) or L-368, 899 (lower; Pre, n = 32; Post, n = 32; recording from 6 mice) application. ns, *p* > 0.05, ***p < 0.001, as determined by paired t-test. Calculation of AUC within the first 10 s of REM sleep to quantify changes in synaptic and neuropeptide activity. The data above are presented as the mean ± SEM. (**K**) GRAB_SST2.0⬌PYR_ biosensor recording in PYR neurons before and after saline (upper; Pre, n = 24; Post, n = 24; recording from 6 mice) or L-368, 899 application (lower; Pre, n = 35; Post, n = 35; recording from 7 mice). ns, *p* > 0.05, ***p < 0.001, as determined by paired t-test. The data above are presented as the mean ± SEM. (**L**) GRAB_VIP1.7⬌PYR_ biosensor recording in PYR neurons before and after saline (upper; Pre, n = 24; Post, n = 24; recording from 5 mice) or L-368, 899 (lower; Pre, n = 32; Post, n = 32; recording from 5 mice) application. ns, *p >* 0.05, ***p < 0.001, as determined by paired t-test. The data above are presented as the mean ± SEM. (**M**) Synaptophysin-Ca^2+^ recording in PV interneurons before and after saline (upper; Pre, n = 18; Post, n = 18; recording from 6 mice) or L-368, 899 (lower; Pre, n = 25; Post, n = 25; recording from 6 mice) application. ns, *p* > 0.05, **p < 0.01, as determined by paired t-test. The data above are presented as the mean ± SEM. **(N)** L-368, 899 application impairs inhibitory balance in PrL microcircuits during REM sleep, characterized by decreased VIP Ca^2+^ activity, VIP release and PV Ca^2+^ activity but increased SST release and PYR Ca^2+^ activity.

In the PrL, the interactions among four major neuron types: pyramidal neurons (PYR), vasoactive intestinal peptide (VIP), somatostatin (SST), and parvalbumin (PV) neurons, have been extensively studied and well-established 16. PV interneurons mainly exert feed-forward inhibitory effects on PYR somas 17, while VIP interneurons preferentially inhibit SST neurons, which in turn primarily synapse onto the dendritic of PYR neurons 18. During REM sleep, a balance exists between somatic inhibition mediated by PV interneurons and dendritic disinhibition mediated by VIP neurons 19. Additionally, VIP interneurons can directly or indirectly (via SST) modulate PV activity 20, contributing to a complex network of inhibitory and disinhibitory interactions. Building on these established connections, we developed a theoretical microcircuit model incorporating three distinct inhibitory balances: (1) VIP and SST neuropeptides regulate dendritic inhibition in PYR neurons during REM sleep; (2) VIP and SST interneurons modulate PV activity to stabilize somatic activity in PYR neurons; and (3) a dynamic balance is maintained between PV-mediated somatic inhibition and VIP-mediated dendritic disinhibition during REM sleep (Figure 4A). This model integrates known circuit mechanisms with new hypotheses about how distinct neuropeptide systems regulate PYR neuron soma and dendrite activity during REM sleep to ensure circuit stability.

We then investigated whether this microcircuit contributes to REM sleep-related social memory consolidation, a process that typically occurs within the first few hours after encoding 12. To explore this, subject mice underwent a 20-minute memory encoding session, during which they explored familiar and novel conspecifics. Following this encoding session, we conducted fiber photometry alongside EEG and EMG recordings to monitor neural activity during the REM sleep consolidation phase (Figure 4B). To examine the role of specific interneurons in this microcircuit, we targeted VIP (a) and PV (d) neurons by expressing rAAV-DIO-synaptophysin-jGCaMP7b in VIP-Cre and PV-Cre mice, enabling us to monitor presynaptic Ca²⁺ activity during REM sleep. Additionally, biosensors specific to SST (b: virus injection of rAAV-CamkII-SST2.0) and VIP (c: via co-injection of of rAAV-CamkII-cre and rAAV-DIO-VIP1.7) neuropeptides were expressed in PYR neurons of WT mice to detect neuropeptide release during REM sleep. These images illustrate that the expression of biosensors and jGCaMP7b in the PrL. Biosensor signals revealed increased VIP neuropeptide release (GRAB_VIP1.7⬌PYR_) during the NREM-to-REM transition, while SST neuropeptide release (GRAB_SST2.0⬌PYR_) remained silent. Simultaneously, VIP and PV interneurons exhibited increased presynaptic Ca²⁺ activity during these transitions, as visualized by jGCaMP7b (Figure 4C-F). This suggests a coordinated role of VIP and PV neurons in stabilizing PYR activity during REM sleep, potentially facilitating social memory consolidation.

Immunostaining revealed that OXT receptors in the PrL were expressed on both excitatory (Suppl Figure S4A) and inhibitory (Suppl Figure S4B-C) neurons. To investigate the role of OXT in modulating PYR activity and inhibitory balance during REM sleep, we administered either an OXT receptor antagonist (L-368,899) or saline in the PrL and assessed neuronal activity before and after treatment (Figure 4G-I). In saline-treated mice, VIP interneurons exhibited robust presynaptic Ca²⁺ activity, effectively suppressing SST neuron activity, as indicated by a reduction in SST neuropeptide release. This suppression underscores the disinhibitory role of VIP neurons on PYR dendrites, while direct VIP-mediated inhibition stabilized dendritic activity. Concurrently, PV neurons exerted inhibitory control over PYR somas, which was consistent with elevated z-scores in presynaptic jGCaMP7b-expressing PV neurons during REM sleep (Figure 4J-M).

In contrast, administration of L-368,899 significantly reduced VIP neuron presynaptic Ca²⁺ activity, resulting in increased SST neuropeptide release and enhanced SST-mediated inhibition of PYR dendrites. Direct VIP-mediated inhibition of PYR dendrites was also attenuated. Furthermore, L-368,899 decreased PV neuron presynaptic Ca²⁺ activity, disrupting somatic inhibition (Figure 4J-M). This impaired inhibitory balance was reflected in enhanced PYR neuron activity during REM sleep, as detected by calcium imaging (Suppl Figure S5A-D), likely due to the weakened inhibitory influence of interneurons on PYR neurons 43. Collectively, these findings demonstrate that OXT modulates both dendritic and somatic inhibition of PYR neurons, maintaining the inhibitory balance necessary for social memory consolidation during REM sleep (Figure 4N). The local L-368,899 application impairs inhibitory balance in PrL microcircuits during REM sleep (Figure 4N).

### Chronic SD impaired REM sleep, OXT release, inhibitory balance in PrL microcircuits during REM sleep and social memory

**Figure 5:**
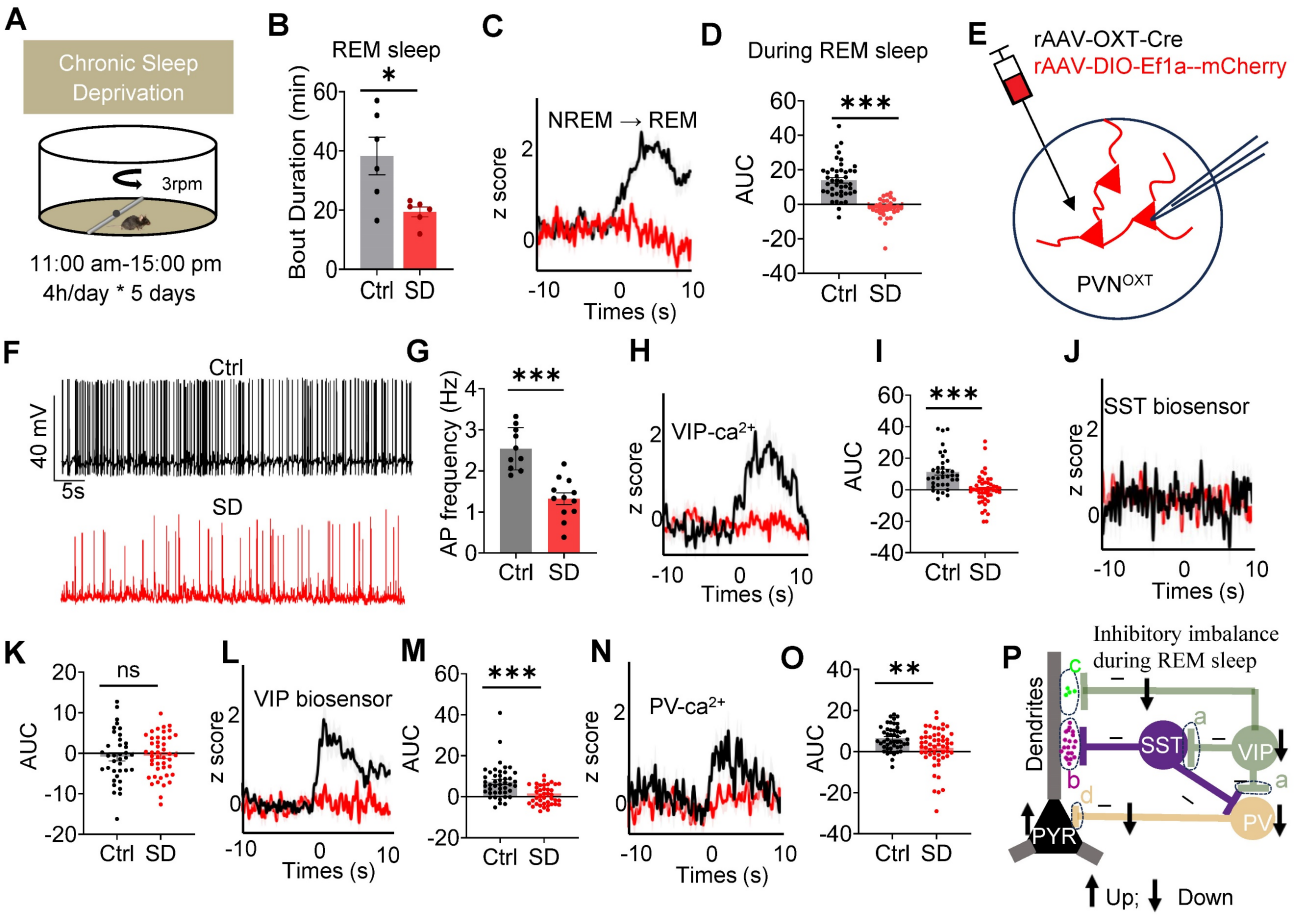
** Chronic SD impaired REM sleep, OXT release and inhibitory balance in PrL.** (**A**) Schematic representation of the chronic SD protocol is provided. (**B**) Comparison of REM sleep duration between the control (Ctrl) and SD group. n = 6 mice per group; *p < 0.05, as determined by unpaired t-test. Data collected from 16:00 pm to 22:00 pm. (**C and D**) Comparison of OXT biosensor fluorescence signal in Prl during REM sleep between ctrl and SD group. Ctrl, n = 44 trials from 4 mice; SD, n = 32 trials from 4 mice; ***p < 0.001, as determined by unpaired t-test. (**E**) Diagram showing whole-cell recording of PVN^OXT^ neurons expressing mix virus rAAV-OXT-Cre and DIO-Ef1a-mCherry with a patch pipette. (**F**) Representative voltage traces recorded from PVN^OXT^ neurons in control and SD groups. (**G**) Chronic SD reduces the spontaneous firing rate of PVN^OXT^ neurons expressing mCherry in the PVN. n=10 cells for the Ctrl group and n=12 cells for the SD group; ***p < 0.001, as determined by unpaired t-test. AP, action potential. (**H**) Synaptophysin-Ca^2+^ recording in VIP interneurons during NREM-to-REM sleep transitions. Black, ctrl; red, SD. (**I**) AUC comparisons of VIP synaptophysin-Ca^2+^ signal. n = 5 mice per group; Ctrl, n = 36 trials from 5 mice; SD, n = 48 trials from 5 mice; ***p < 0.001, as determined by unpaired t-test. (**J**) GRAB_SST2.0⬌PYR_ biosensor recording during NREM-to-REM sleep transitions. (**K**) AUC comparisons of GRAB_SST2.0⬌PYR_ biosensor activity. Ctrl, n = 37 trials from 7 mice; SD, n = 48 trials from 7 mice; ns, *p* > 0.05, as determined by unpaired t-test. (**L**) GRAB_VIP1.7⬌PYR_ biosensor recording during NREM-to-REM sleep transitions. (**M**) AUC comparisons of GRAB_VIP1.7⬌PYR_ biosensor activity. Ctrl, n = 37 trials from 5 mice; SD, n = 48 trials from 3 mice; ns, ***p < 0.001, as determined by unpaired t-test. (**N**) Synaptophysin-Ca^2+^ recording in PV interneurons during NREM-to-REM sleep transitions. (**O**) AUC comparisons of PV synaptophysin-Ca^2+^ activity. Ctrl, n = 47 trials from 6 mice; SD, n = 54 trials from 6 mice; **p < 0.01, as determined by unpaired t-test. The data above are presented as the mean ± SEM. (**P**) Chronic SD disrupts inhibitory balance in PrL microcircuits during REM sleep, characterized by decreased VIP Ca^2+^ activity, VIP release and PV Ca^2+^ activity but increased PYR Ca^2+^ activity.

To assess the effects of chronic SD on social memory, we established a chronic SD mouse model and evaluated social memory performance (Suppl Figure S6A-B). SD mice exhibited significant social memory deficits (Suppl Figure S6C-D) accompanied by a reduction in REM sleep duration (Figure 5A-B). Additionally, a reduction in OXT release in the PrL during REM sleep was observed in SD mice (Figure 5C-D). An *in vitro* study demonstrated that chronic SD reduced the spontaneous firing rate of PVN^OXT^ neurons (Figure 5E-G), providing direct evidence that impaired PVN^OXT^ neurons activity contributes to the reduced OXT release in PrL.

To further investigate the impact of chronic SD on the inhibitory balance in PrL microcircuits during REM sleep, we analyzed presynaptic Ca²⁺ activity and neuropeptide release in these circuits. rAAV-DIO-synaptophysin-jGCaMP7b was injected into the PrL of VIP-Cre and PV-Cre mice to monitor presynaptic Ca²⁺ activity during REM sleep. Additionally, SST and VIP biosensors were expressed in the PYR neurons of WT mice to assess neuropeptide release. In SD mice, VIP neurons exhibited decreased presynaptic Ca²⁺ activity, resulting in reduced VIP neuropeptide release. Similarly, presynaptic Ca²⁺ activity in PV neurons was also diminished (Figure 5H-O). Additionally, Ca²⁺ imaging revealed elevated Ca²⁺ signaling in PYR neurons during REM sleep in SD mice (Suppl Figure S7A-D). These results align with previous studies showing impaired excitability of PV interneurons and increased PYR neuron activity in the early stages of an AD mouse model 43. Together, these results indicate that chronic SD disrupts the balance between VIP-mediated dendritic disinhibition and PV-mediated somatic inhibition. Specifically, SD reduces dendritic inhibition and weakens somatic inhibition, leading to a pronounced imbalance in PYR neuron activity during REM sleep (Figure 5P). Such disruptions likely contribute to the observed impairments in social memory consolidation.

### REM sleep-selective activation of PVN^OXT^-PrL pathway or intranasal OXT administration alleviated social memory deficits in SD mice

Given reduced OXT release in PrL are strongly associated with dysfunction of PVN^OXT^ neurons, we hypothesized that enhancing PVN^OXT^ neuron activity could could restore OXT levels in the PrL. To test this, we injected rAAV-OXT-Cre and Cre-dependent rAAV-ChR2 or rAAV-mCherry into the bilateral PVN and implanted an optical fiber in the PrL to monitor OXT levels via the GRAB sensor. Upon activation of PVN^OXT^ neurons, OXT release in the PrL was significantly increased in the ChR2 group compared with the mCherry group in SD mice (Suppl Figure S8A-C).

**Figure 6:**
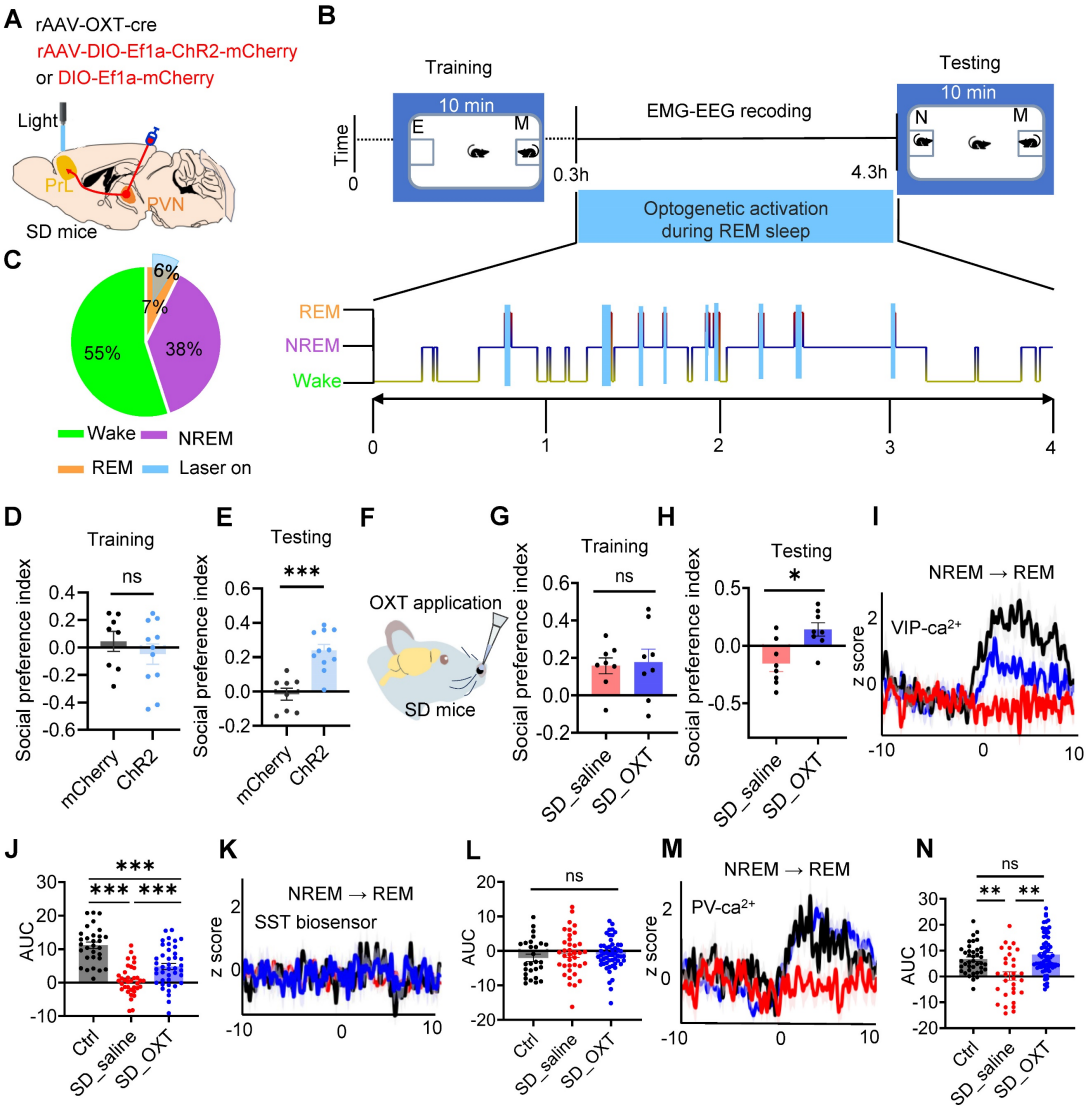
** REM sleep-selective activation of PVN^OXT^-PrL pathway or intranasal OXT administration alleviated social memory deficits in SD mice.** (**A**) Diagram showing bilateral viral injection site of rAAV-OXT-cre, rAAV-DIO- ChR2-mCherry/rAAV-DIO-mCherry in unilateral PVN, optical fiber implanted above PrL, EEG recording in cortex and EMG recording in neck muscles. (**B**) Protocol outline for optogenetic manipulation and two-choice social novelty test. E, empty; M, mice; N, novel mice; F, familiar mice. (**C**) Total duration of sleep-wake state during optogenetic manipulation in PVN^OXT^-PrL circuit. (**D and E**) Optogenetic activation of PVN^OXT^-PrL pathway during REM sleep improved social memory in SD mice as assessed by two-choice social novelty test. Social preference index was measured in training (K) and testing (L) phase, respectively. n = 8 mice in mCherry group; n = 11 mice in ChR2 group; ns, *p >* 0.05, ***p < 0.001, as determined by unpaired t-test. (**F to H**) Intranasal administration of OXT improved social momery in SD mice as assessed by two-choice social novelty test. Social preference index was measured in training (G) and testing (H) phase, respectively. n = 8 mice per group; ns, *p* > 0.05, *p < 0.05, as determined by unpaired t-test. (**I**) Synaptophysin-Ca^2+^ recording in VIP interneurons during NREM-to-REM sleep transitions (black, ctrl; red, SD_saline; blue, SD_OXT). (**J**) AUC comparisons of VIP synaptophysin-Ca^2+^ activity. n = 5 mice per group; Ctrl, n = 30 trials; SD_saline, n = 36 trials; SD_OXT = 41 trials; ***p < 0.001, as determined by One-way ANOVA. (**K**) GRAB_SST2.0⬌PYR_ biosensor recording during NREM-to-REM sleep transitions. (**L**) AUC comparisons of GRAB_SST2.0⬌PYR_ biosensor activity. Ctrl, n = 27 trials from 5 mice; SD_saline, n = 37 trials from 7 mice; SD_OXT, n = 53 trials from 4 mice; ns, *p* > 0.05, as determined by One-way ANOVA. (**M**) Synaptophysin-Ca^2+^ recording in PV interneurons during NREM-to-REM sleep transitions. (**N**) AUC comparisons of PV synaptophysin-Ca^2+^ activity. Ctrl, n = 40 trials from 8 mice; SD_saline, n = 30 trials from 8 mice; SD_OXT, n = 69 trials from 4 mice; ns, *p* > 0.05; **p < 0.01, as determined by One-way ANOVA. The data above are presented as the mean ± SEM.

To determine whether activating the PVN^OXT^-PrL pathway during REM sleep could rescue SD-induced social memory deficits, we selectively stimulated this projection using axonal projection-specific optogenetics. Bilateral injections of rAAV-OXT-Cre and Cre-dependent rAAV-ChR2-mCherry or rAAV-mCherry were performed in the PVN of WT mice, followed by implantation of fiber optics and EEG-EMG devices (Figure 6A). We selectively activated this projection during REM sleep and evaluated its impact on social memory using a two-choice social memory test (Figure 6B). Blue light stimulation was applied during approximately 90% of cumulative REM sleep in both groups (Figure 6C). While PVN^OXT^-PrL pathway activation slightly increased NREM bout numbers, no significant changes were observed in other aspects of sleep architecture (Suppl Figure S9). During the training phase, both groups exhibited comparable social preference (Figure 6D). However, in the testing phase, the ChR2 group showed significantly improved social memory, as demonstrated by a higher social novelty preference index (Figure 6E). These results indicate that selective activation of the PVN^OXT^-PrL pathway during REM sleep effectively ameliorates social memory deficits caused by SD.

Intranasal administration of OXT, a non-invasive and convenient approach, has been shown to elevate central OXT levels and modulate cognitive and memory functions 44. Given the reduction in OXT release within the PrL during REM sleep in SD mice, we assessed whether intranasal OXT administration could alleviate SD-induced social memory deficits. Intranasal OXT significantly restored social memory performance in SD mice (Figure 6F-H).

We next investigated whether intranasal OXT could restore the inhibitory imbalance in PrL microcircuits during REM sleep. rAAV-DIO-synaptophysin-jGCaMP7b was injected into the PrL of VIP-Cre and PV-Cre mice to monitor presynaptic Ca²⁺ activity during REM sleep. Additionally, SST and VIP biosensors were expressed in the PYR neurons of WT mice to assess neuropeptide release within PrL microcircuits during REM sleep. Following OXT administration, presynaptic Ca²⁺ activity in VIP and PV neurons increased significantly, reaching levels comparable to control mice, while GRAB_SST2.0⬌PYR_ biosensor signals remained silent during NREM-to-REM transitions (Figure 6I-N). It is known that SST neuron somatic activity is typically elevated upon waking 19. Interestingly, following OXT treatment, SST neuropeptide release was increased, suggesting that this activity was partially restored (Suppl Figure S10A-C). Although SST activity had declined due to chronic SD, it exhibited partial recovery following OXT treatment. These findings suggest that intranasal OXT restores the inhibitory balance in PrL microcircuits during REM sleep by enhancing VIP-mediated dendritic disinhibition and PV-mediated somatic inhibition of PYR neurons. This recovery contributes to the amelioration of social memory impairments in SD mice.

## Discussion

The mechanisms by which OXT modulates PrL microcircuits to support social memory consolidation are not fully understood. In this study, we identify the PVN^OXT^-PrL pathway and OXT release during REM sleep as critical regulators of social memory consolidation. Our findings demonstrate that OXT release during REM sleep stabilizes the balance between VIP-mediated dendritic disinhibition and PV-mediated somatic inhibition, thereby facilitating memory consolidation. Disruption of this balance, whether by local OXT receptor antagonism or SD, impairs social memory. Selective activation of the PVN^OXT^-PrL pathway or intranasal OXT administration restores inhibitory balance and rescues social memory deficits in SD mice. These findings highlight the essential role of OXT in regulating PrL microcircuits during REM sleep to support social memory.

NREM sleep and REM sleep contribute to memory reinforcement and memory refinement, respectively 3. Reactivation of learning-associated neural circuits during NREM sleep promotes memory consolidation 8, including social memory 45. Recent findings have also implicated that SuM to CA2 are required for the consolidation of social memory during REM sleep 13. OXT, synthesized in the PVN and supraoptic nuclei, is critical for social behaviors and memory 46. The PVN synthesize OXT and transport it for release into dendrites and axons. Oxytocinergic axons are widely distributed throughout the brain, including the thalamus, cortex, amygdala and hippocampus 46. OXT acts on CA2 47, 48 or SUM 23 to modulate social memory. The PrL is rich in OXT receptors 49 and modulates the consolidation of memory 50-52. Some evidence suggested that OXT is crucial for memory consolidation 53, 54. Consistent with these findings, our results demonstrate that inhibiting the PVN^OXT^-PrL pathway or blocking OXT receptors in the PrL impairs REM sleep and social memory. Conversely, activation of this pathway during REM sleep restores OXT release and rescues social memory. These findings highlight the critical role of OXT in the PrL for social memory consolidation and underscore its therapeutic potential for memory impairments.

During REM sleep-related emotional memory consolidation, VIP and PV neurons in PFC exhibit increased activity, while SST neurons remain silent. The balance between PV-mediated somatic inhibition and VIP-mediated dendritic disinhibition is crucial for emotional memory consolidation 19. Using presynaptic calcium imaging combined with multiple biosensors, we observed a similar balance during social memory consolidation. Notably, we found that reduced OXT signaling decreased the activity of both VIP and PV neurons, thereby disrupting the inhibitory balance within PrL microcircuits during REM sleep. These findings provide novel insights into the role of OXT in regulating social memory consolidation during REM sleep. This balance appears to facilitate social memory consolidation by enabling dendritic disinhibition to create a temporal window for reinforcing wakeful experiences while somatic inhibition prevents excessive top-down reinforcement of memory traces. A lack of somatic inhibition may disrupt the local dendritic computation through reverse propagation activity 55, 56. Such imbalances could lead to the over-consolidation of emotionally salient memories, as observed in conditions like PTSD and REM sleep-related affective disorders 57, 58. The coordinated processing of dendritic and somatic inputs in pyramidal neurons enhances their information-processing capacity, supporting complex cognitive functions, including memory consolidation. Our results show that inhibitory imbalance caused by OXT receptor antagonism or chronic SD increases pyramidal neuron activity, resembling early pathology in AD 43. We hypothesize that REM sleep disruption and OXT deficiency in the PrL play a role in social memory deficits in AD.

OXT signaling is implicated in diverse cognitive functions, including spatial and working memory 59. Cholecystokinin (CCK) neurons in PFC are involved in working memory retrieval 60. We hypothesize that OXT may modulate social memory retrieval by affecting CCK neuron activity in the PrL, a hypothesis that warrants further exploration. While OXT's effects on spatial and episodic memory are primarily mediated by the hippocampal CA2 region 61, its role in working memory likely involves stress regulation and executive processes within other PFC regions 62. These findings suggest that the PVN^OXT^-PrL pathway specifically supports social memory consolidation, and future research should explore potential interactions with circuits underlying spatial and working memory. This could elucidate whether OXT's influence on social memory is uniquely specialized.

Consistent with previous studies, our findings show that chronic SD reduces OXT levels in the brain 63, as evidenced by decreased GRABOXT1.8 fluorescence in the PrL of SD mice, reflecting diminished OXT regulation. SD also alters dendritic length and branching in pyramidal neurons in the cerebral cortex 27, which may contribute to social memory deficits. Moreover, chronic SD impairs inhibitory circuits, including VIP and PV neurons, offering potential insights into the pathophysiology of disorders such as autism spectrum disorder (ASD) and schizophrenia, where disrupted cortical inhibitory-excitatory balance is a hallmark 64. Further studies on the impact of chronic SD on OXT receptor function and OXT release could inform therapeutic strategies to restore inhibitory-excitatory balance in these conditions.

This study has several limitations that warrant acknowledgment. Firstly, we employed a chronic SD model to disrupt both NREM and REM sleep and evaluate their effects on social memory consolidation. While the flowerpot method for REM-specific SD 65 was initially considered, the associated stress responses significantly confounded behavioral outcomes. To minimize stress and better simulate clinically relevant sleep disturbances, we adopted a chronic SD approach that disrupts overall sleep. However, this approach limits our ability to isolate the specific contributions of REM sleep. Future studies using automated systems for targeted REM sleep disruption are essential to clarify its distinct role in memory consolidation. Secondly, the OXT-promoter-driven viral construct used in this study demonstrated limited specificity and efficacy, achieving approximately 66% efficiency (Supplementary Figure S11A-B). The use of OXT-Cre mice in future research could enhance the precision and reliability of experimental outcomes. Third, the widespread expression of OXT receptors across diverse neuronal subtypes 66, 67 poses challenges in isolating their specific effects on pyramidal, VIP, and PV neurons. Further research is necessary to delineate the independent roles of OXT in modulating these neuronal populations. Finally, although the PVN is the primary source of OXT secretion, PVN^OXT^ neurons also project to other brain regions, such as thalamus, amygdala and hippocampal CA2. Investigating the interactions between these regions and the PrL will be critical for understanding their collaborative roles in social memory consolidation. Addressing these limitations in future studies will enhance our understanding of the mechanisms underlying OXT-mediated regulation of neural circuits.

## Conclusions

Our study identifies the PVN^OXT^-PrL pathway as essential for REM sleep-dependent social memory consolidation, mediated by OXT. By maintaining the balance between VIP-mediated dendritic disinhibition and PV-mediated somatic inhibition, OXT ensures proper microcircuit function in the PrL. Disruption of this balance, through OXT receptor antagonism or sleep deprivation, impairs social memory, while pathway activation or intranasal OXT rescues these deficits. These findings show a novel mechanism by which OXT supports social memory, offering insights into its role in social behavior and highlighting potential therapeutic targets for disorders involving social memory deficits. Moreover, our results underscore the importance of REM sleep as a critical window for OXT-mediated neuroplasticity, with implications for understanding and treating sleep-related cognitive and emotional disorders.

## Supplementary Material

Supplementary figures and table.
