# Supplementary material for: Oxytocin modulates inhibitory balance in the prelimbic cortex to support social memory consolidation during REM sleep
Source: Theranostics. 2025 Feb 18;15(8):3257–74. doi: 10.7150/thno.109104 (PMC11905142; doi:10.7150/thno.109104)
Supplement: Supplementary file 1 — Supplementary figures and table. [file thnov15p3257s1.pdf]

Oxytocin modulates inhibitory balance in the prelimbic cortex to support social memory consolidation during REM sleep

[†]Yanchao Liu et al. Email: yanchao_liu@whu,edu.cn
[‡]Corresponding author. Email: zqp005098@whu.edu.cn (Q.-P.Z.); gaoyangbest@whu.edu.cn (Y.G.); xuhaibo@whu.edu.cn (H.-B.X.)

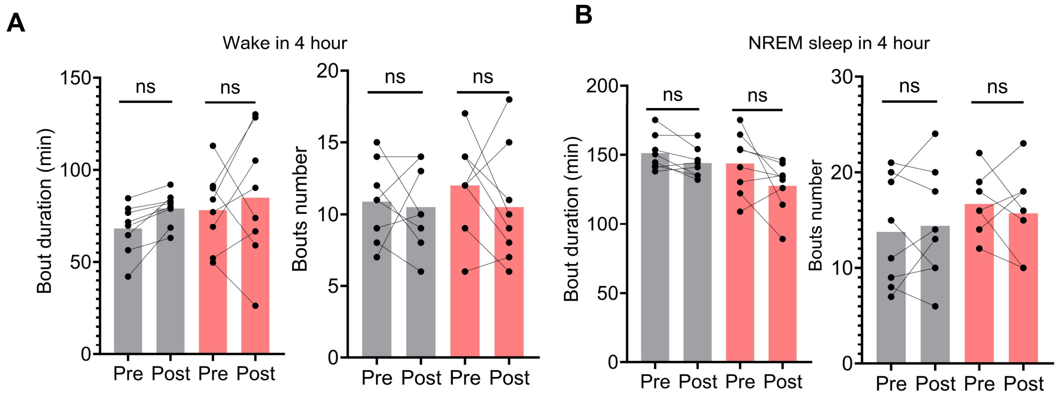

Figure S1. OXT receptor antagonists did not affect NREM sleep and wake. (**A and B**) Bilateral antagonism of OXT receptors in PrL did not affect wake (A) and NREM sleep (B) in mice. n = 8, ns, $p > 0.05$, as determined by unpaired t-test. Data are expressed as mean ± SEM.

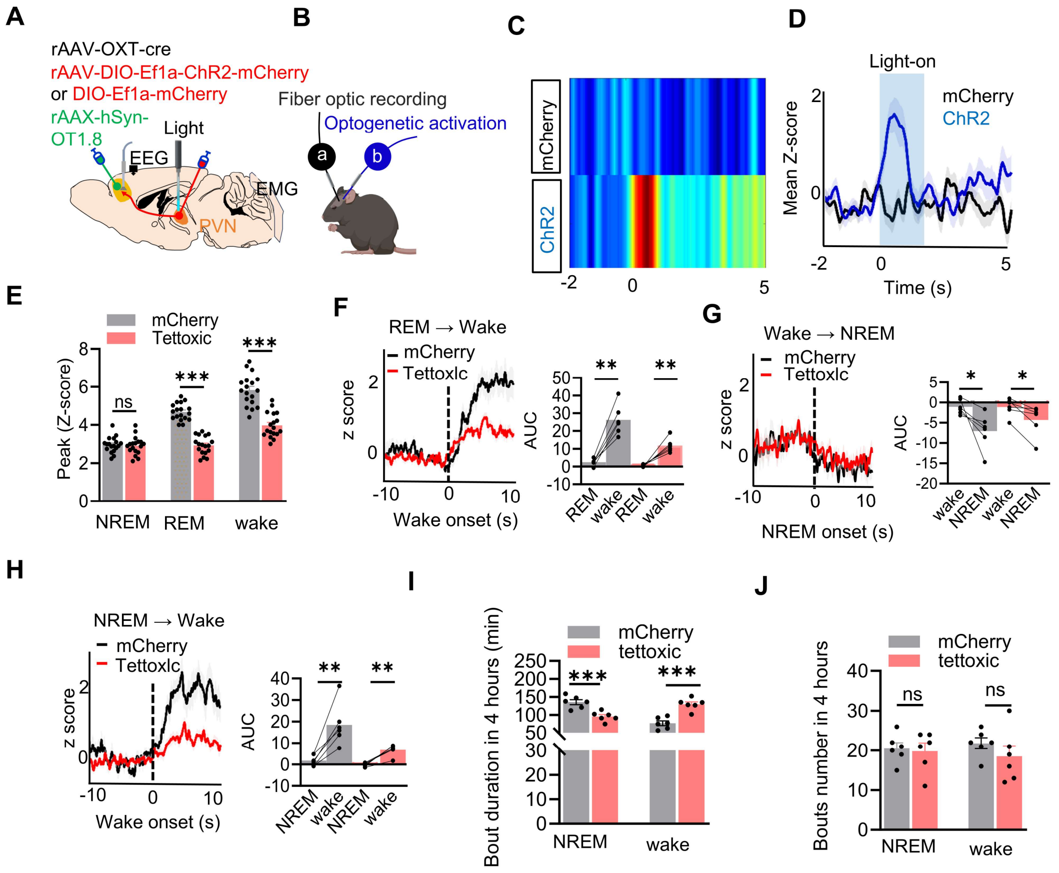

Figure S2. Population activity of PVN^{OXT} neurons affected OXT release in PrL, wake and NREM sleep.

(**A to D**) Schematic of optogenetics virus injection, photostimulation and fluorescence recordings. Peri-event plots illustrate the averaged fluorescence z scores of mcherry group (n = 4) and ChR2 group (n = 4) in response to photostimulation of PVN^{OXT} neurons (473 nm laser, a train of ten 10-ms light pulses at 10 HZ, 1s on and 50 s off for 20 min, blue vertical bars). The curves and shaded regions indicate the mean ± SEM.

(**E**) Comparison of peak OXT biosensor fluorescence signal during wake, NREM sleep, and REM sleep in mCherry and tettoxlc group. n=18, three sessions per mouse from 6 mice; ns, $p > 0.05$; ***$p < 0.001$, as determined by unpaired t-test.

(**F to H**) OXT biosensor fluorescence signal transformation aligned to sleep-wake state transitions. Comparison of AUC over 10 s during wake, NREM, and REM sleep. *$p < 0.05$; **$p < 0.01$, as determined by paired t-test.

(I and J) Duration and bouts of NREM Sleep and wake over a 4-hour in two groups of

mice. ns, $p > 0.05$; $***p < 0.001$, as determined by unpaired t-test.

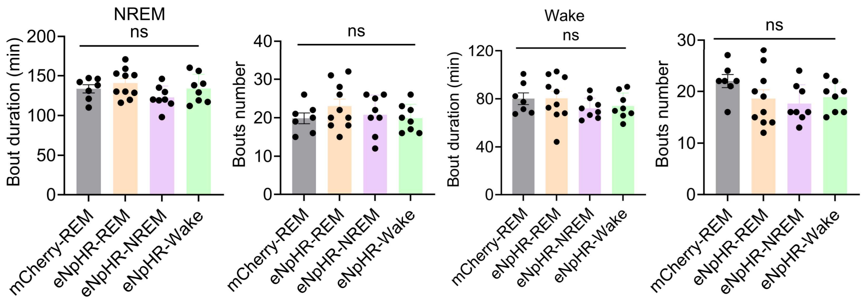

Figure S3. Photoinhibition of PVN^{OXT}-PrL pathway during REM sleep/NREM sleep/wake phase did not affect NREM sleep and wake.

mCherry-REM group, n = 7 mice; eNpHR-REM group, n = 10 mice; eNpHR-NREM and eNpHR-Wake groups, n = 8 mice each; ns, $p > 0.05$, as determined by unpaired t-test. Data are expressed as mean ± SEM.

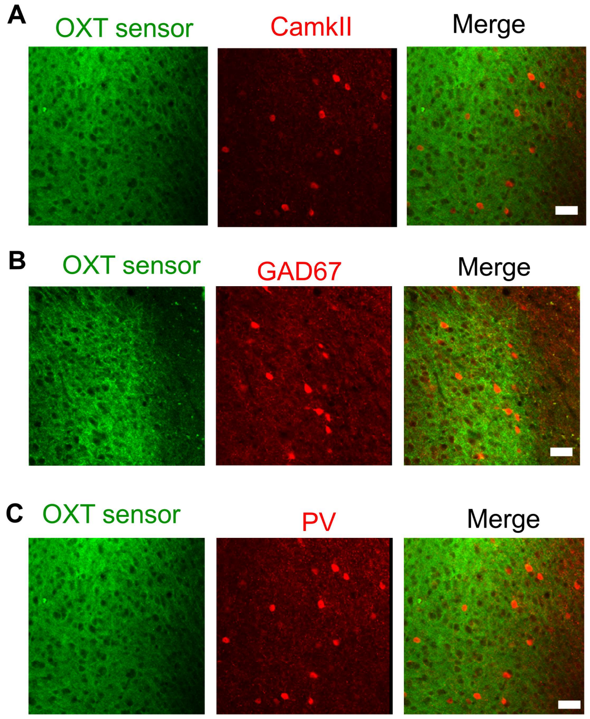

Figure S4. OXT receptor distribution on excitatory and inhibitory neurons.

A. Representative photomicrograph OXT sensor in PrL (left, green), CamkII immunolabeling (middle, red) and merged image (right). n = 2 mice. Scale bar = 200 µm.

B. Fluorescence images of OXT sensor in PrL (left, green), immunostaining of GAD67

(middle, red) and merged image (right). n = 2 mice. Scale bar = 200 μm.

C. Representative image of OXT sensor in PrL (left, green), PV immunolabeling (middle, red) and merged image (right). n = 2 mice. Scale bar = 200 μm.

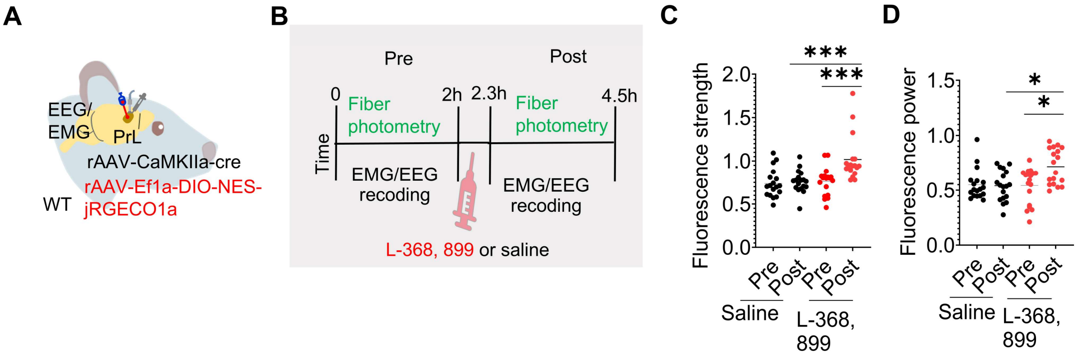

Figure S5. Higher Ca^{2+} activity in pyramidal neurons was observed during REM sleep after local OXT receptor antagonism treatment in PrL.

(**A**) Diagram illustrating virus injection, cannula placement, setup for fiber photometry and EMG/EEG recording in mice.

(**B**) Timeline showing administration of L-368, 899 (OXT receptor antagonist) or saline.

(**C and D**) Comparison of fluorescence strength (C), fluorescence power (D) of PYR neurons Ca^{2+} signal before and after application of L-368, 899 or saline during REM sleep. n=18, three sessions per mouse from 6 mice; **p* < 0.05; ****p < 0.001*, as determined by paired and unpaired t-test.

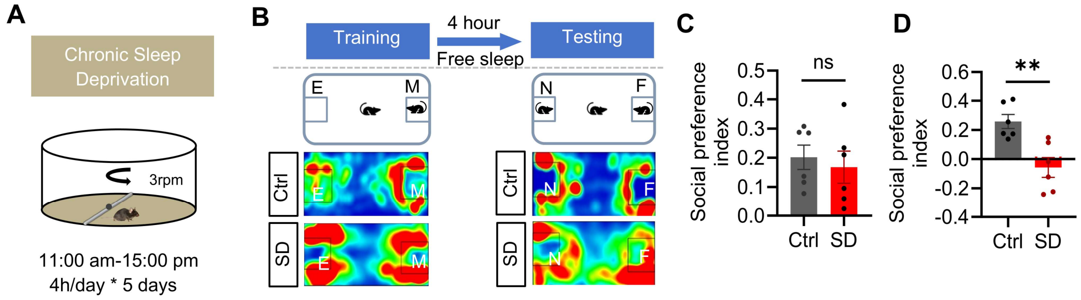

Figure S6. Chronic SD impaired social memory in mice.

(**A**) Protocol for chronic SD.

(**B**) Upper, two-choice social memory test. E, empty; M, mice; N, novel mice; F, familiar mice. Lower, representative heatmaps of distribution of time in two-choice task.

(**C and D**) Social preference index was assessed by two-choice social novelty test in training (C) and testing (D) phase, respectively. n = 6 mice; ns, *p > 0.05*; ***p < 0.01*,

as determined by unpaired t-test.

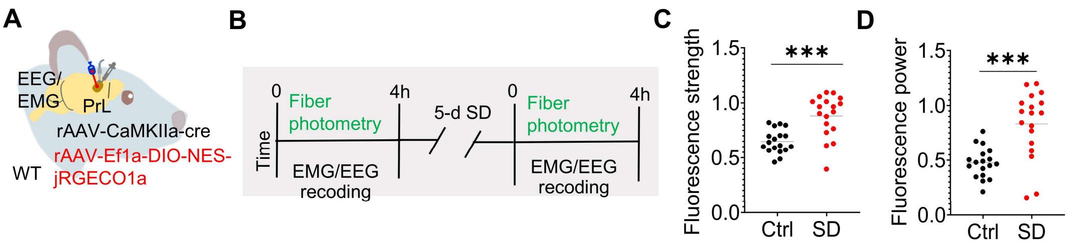

Figure S7. Higher Ca^{2+} activity in pyramidal neurons in PrL was observed during REM sleep in chronic SD mice.

(**A**) Diagram illustrating virus injection, setup for fiber photometry and EMG-EEG recording in mice.

(**B**) Schematic of Fiber photometry and EMG-EEG recording.

(**C and D**) Comparison of fluorescence strength (C), fluorescence power (D) of PYR neurons Ca^{2+} signal during REM sleep between Ctrl and SD group. n = 18, three sessions per mouse from 6 mice; ****p < 0.001*, as determined by unpaired t-test.

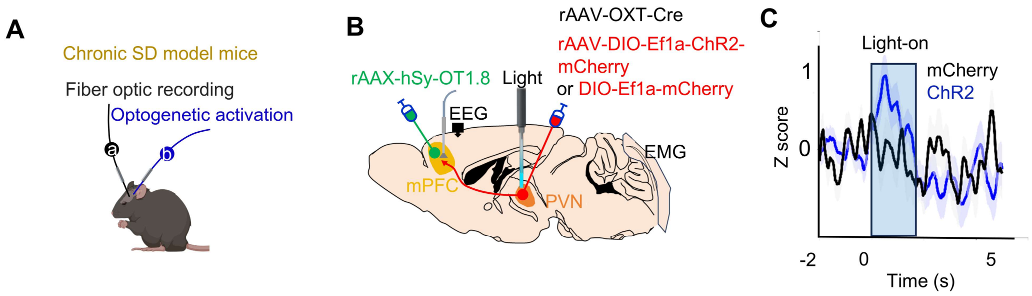

Figure S8. OXT fluorescence in PrL increased after the activation of PVN^{OXT} neurons in SD mice.

(**A and B)** Schematic of optogenetics virus injection, photostimulation and fluorescence recordings in SD mice.

(C) OXT fluorescence in PrL increased after the activation of PVN^{OXT} neurons in SD compared with mCherry (n = 4, 473 nm laser, a train of ten 10-ms light pulses at 10 Hz, 1 s-on and 50 s-off for 20 min, blue vertical bars). The curves and shaded regions indicate the mean ± SEM.

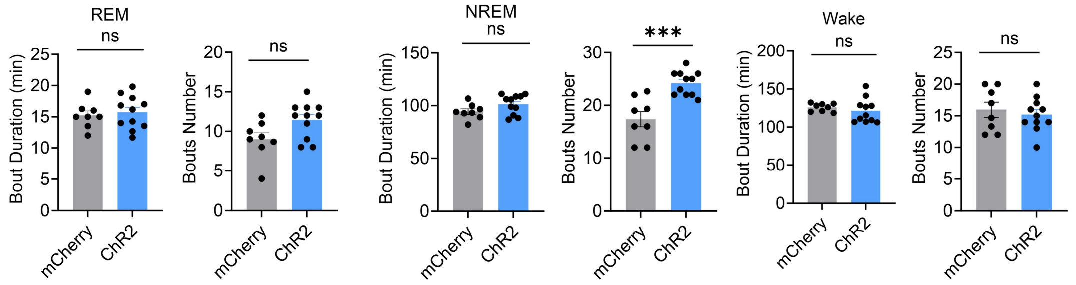

Figure S9. Optogenetic activation of PVN^{OXT}-PrL pathway during REM sleep did not affect sleep and wake duration in SD mice.

Photoactivation of the PVN^{OXT}-PrL pathway during REM sleep could affect sleep-wake with a slightly higher number of REM and NREM occurrences. n = 8 mice in mCherry group; n = 11 mice in ChR2 group; ns, *p > 0.05; *p < 0.05*; ***p < 0.01*, as determined by unpaired t-test.

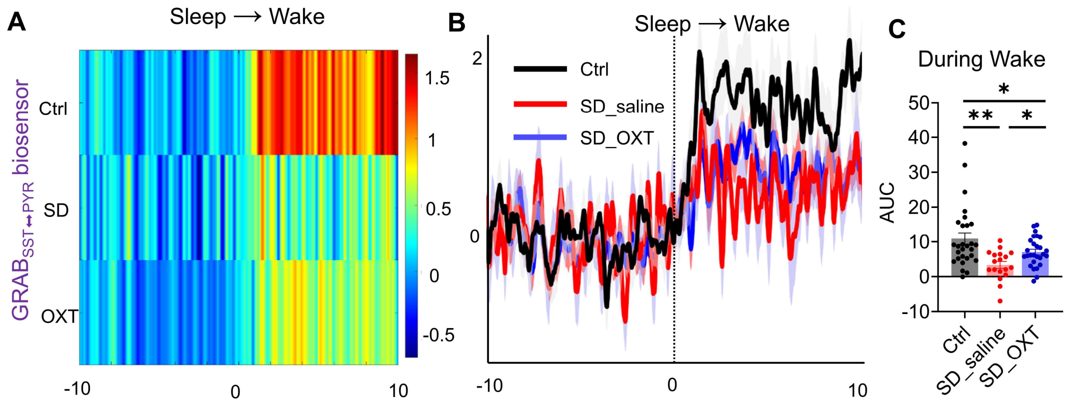

Figure S10. Intranasal OXT restored reduced SST release in PrL in SD mice.

(A) Individual transitions with color-coded fluorescence intensity from sleep to wake in three groups.

(B) Mean ± SEM activity profiles of GRAB$_{SST\leftrightarrow PYR}$ biosensor in PrL during the transition from sleep to wake. (black = ctrl, red = SD_saline, blue = SD_OXT).

(C) AUC comparisons of GRAB$_{SST2.0\leftrightarrow PYR}$ biosensor activity in PrL during wake. Ctrl, n = 28 trials from 5 mice; SD_saline, n = 18 trials from 4 mice; SD_OXT, n = 27 trials from 4 mice; *$p < 0.05$, **$p < 0.01$, as determined by One-way ANOVA.

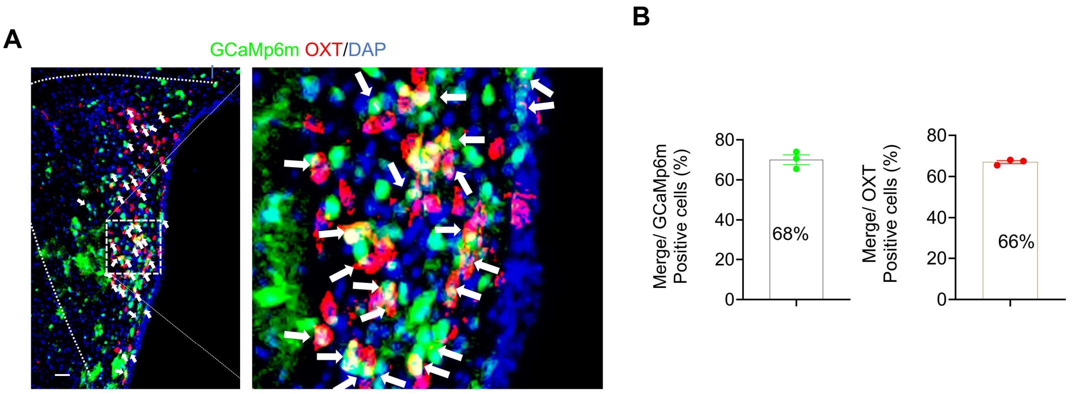

Figure S11. The specificity and efficiency of the OXT-promoter-driven virus construct.

(**A**) Overlap between GCaMP6m and immunostaining of OXT in the PVN. Representative photomicrographs of PVN^{OXT} neurons from a mouse microinjected with rAAV-OXT-Cre and AAV-DIO-hSyn-GCaMP6m at the PVN. The GCaMP6m (green) and OXT immunolabeling (red) indicate GCaMP6m and OXT-expressing neurons, respectively, and the yellow image depicts merged neurons. Scale bar = 200 µm.

(B) Percentage of Gcamp6m (green)/OXT double-positive cells versus Gcamp6m positive cells (left) or versus OXT-positive cells (right). n = 3 mice.

KEY RESOURCES TABLE

**REAGENT or RESOURCE**	SOURCE	IDENTIFIER
**Antibodies**		
Alexa Fluor 546 donkey anti-rabbit	Servicebio	GB21303
Alexa Fluor 546 donkey anti-mouse	Servicebio	GB21301
mouse anti-CamKII	Cell signaling	3362
mouse anti-GAD67	Sigma	MAB5406
mouse anti-Parvalbumin	Sigma	SAB4200545
rabbit anti-Oxytocin-neurophysin 1	abcam	EPR20973

**Virus**		
rAAV9-hSyn-OT1.8	Brain case Co., Ltd.	Cat#BC-1119
rAAV2/9-camkII-SST2.0	BrainVTACo.,Ltd.	Cat#PT-7175
rAAV2/9-DIO-VIP1.7	BrainVTACo.,Ltd.	Cat#PT-8304
rAAV-CaMKIIa-CRE-WPRE-hGH polyA	BrainVTACo.,Ltd.	Cat#PT-0220
Raav-EF1a-DIO-NES-jRGECO1a	Brain case Co., Ltd.	Cat#BC-0212
rAAV2/9-OXT-Cre-WPRE-hGH-pA	BrainVTACo.,Ltd.	Cat#PT-6086
rAAV2/9-CAG-DIO-axon-jGCaMP7b	BrainVTACo.,Ltd.	Cat#PT-8161
rAAV-EF1a-DIO-synaptophysin-jGCaMP7b	Brain case Co., Ltd.	Cat#BC-1378
rAAV2/9-DIO-EF1a-hChR2 (H134R)-mCherry	BrainVTACo.,Ltd.	Cat#PT-3787
rAAV2/9-DIO-EF1a-eNpHR3.0-mCherry	BrainVTACo.,Ltd.	Cat#PT-0007
rAAV2/5- EF1a-DIO-tettoxicP2A-mcherry	BrainVTACo.,Ltd.	Cat#PT-2139
rAAV-EF1a-DIO-GCaMp6m-WPRE-hGH polyA	BrainVTACo.,Ltd.	Cat#PT-0283
rAAV2/9-DIO-Ef1a-mCherry	BrainVTACo.,Ltd.	Cat#PT-0115
**Animals**		
Mouse: C57BL/6J	Beijing Vital River	SCXK：2022-0030

	Laboratory Animal Technology Co., Ltd.	
Mouse: PV-Cre (C57BL/6)	Beijing Vital River Laboratory Animal Technology Co., Ltd.	Gifted by Professor Jianzhi Wang's research group
Mouse: VIP-Cre (C57BL/7)	Genepax Biotechnology Co., Ltd	GAP1043

112
